# Laboratory and Clinical Evaluation of DNA Microarray for the Detection of Carbapenemase Genes in Gram-Negative Bacteria from Hospitalized Patients

**DOI:** 10.1155/2019/8219748

**Published:** 2019-05-13

**Authors:** Yi Song, Fengna Dou, Sha He, Yu Zhou, Qiqi Liu

**Affiliations:** ^1^Beijing Institute of Radiation Medicine, Beijing, China; ^2^Beijing Key Laboratory of New Molecular Diagnosis Technologies for Infectious Diseases, Beijing, China; ^3^The First Hospital of Hebei Medical University, Hebei, China; ^4^Clinical Laboratory of Second Medical Center of PLA General Hospital, Beijing, China

## Abstract

**Background:**

The prevalence of a variety of carbapenemases in Gram-negative bacteria (GNB) has posed a global threat on clinical control and management. Monitoring and controlling the carbapenemase-producing GNB became imperative tasks for many healthcare centers. The aim of this study was to develop a high-throughput, specific, sensitive, and rapid DNA microarray-based method for the diagnosis, phenotypic confirmation, and molecular epidemiological study of carbapenemase genes.

**Methods:**

We targeted a panel of eight carbapenemase genes, including *bla*_KPC_, *bla*_NDM-1_, *bla*_OXA-23_, *bla*_OXA-48_, *bla*_OXA-51_, *bla*_IMP_, *bla*_VIM_, and *bla*_DIM_ for detection. Ultrasensitive chemiluminescence (CL) detection method was developed and used to simultaneously detect eight carbapenemase genes, and plasmids were established as positive or limit of detection (LOD) reference materials. Antibiotic susceptibility was determined by disk diffusion according to Clinical and Laboratory Standards Institute (CLSI) guidelines in order to screen clinical isolates resistant to carbapenem antibiotics as well as Sanger sequencing which was used to confirm the reliability of the results presented by DNA microarray.

**Results:**

Eight carbapenemase genes could be detected with high sensitivity and specificity. The absolute LOD of this strategy to detect serially diluted plasmids of eight carbapenemase genes was 10^2^- 10^3^copies/*μ*L. Then, 416 specimens collected from hospital were detected and the results showed 96.6% concordance between the phenotypic and microarray tests. Compared with Sanger sequencing, a specificity and sensitivity of 100% were recorded for *bla*_NDM-1_, *bla*_IMP_, *bla*_VIM_, and *bla*_DIM_ genes. The specificity for *bla*_KPC_, *bla*_OXA-23_, *bla*_OXA-48_, and *bla*_OXA-51_ genes was 100% and the sensitivity was 98.5%, 97.6%, 95.7%, and 97.9%, respectively. The overall consistency rate between the sequencing and microarray is 97.8%.

**Conclusions:**

The proposed ultrasensitive CL imaging DNA hybridization has high specificity, sensitivity, and reproducibility and could detect and differentiate clinical specimens that carried various carbapenemase genes, suggesting that the method can conveniently be customized for high-throughput detection of the carbapenemase-producing GNB and can be easily adapted for various clinical applications.

## 1. Introduction

Carbapenems are a class of *β*-lactam antibiotics with a broad spectrum and served as the last line against ESBLs efficiently and stably [1]. With wide and heavy use of various antibiotics, carbapenem-resistant isolates have become worldwide public-health issue with a widespread distribution, broad range of activities against *β*-lactams, and increased patient morbidity, mortality, and lengths of hospital stay [2], particularly among elderly patients, infants, and patients with severe underlying disease. In the clinic, it is imperative to develop a rapid, simple, and accurate test to detect and identify the clinical strains that produce carbapenemase, and this is critical for the management and control of the increasing prevalence of carbapenemase-producing strains worldwide [3].

The detection of carbapenemase producers in clinical specimens is based first on the analysis of susceptibility testing according to CLSI updated in 2017 [4], followed by confirmatory genotypic tests with methods like PCR and mass spectrometry [3, 5]. However, these tests are not the best fit for detecting the carbapenemase-producing strains with desirable specificity and sensitivity [6]. Even the recommended CLSI methods are limited by their inherent disadvantages: the phenotypic tests for carbapenemase-producing strains suffer problems of false positives and false negatives [5, 7]. Mass spectrometry cannot provide molecular epidemiological information and is cost-ineffective. Addressing these deficiencies, we herein propose a novel DNA microarray-based method for rapid, sensitive, and specific detection of clinical carbapenamase-producing samples. The proposed assay may have important implications in the diagnosis, phenotypic confirmation, and the molecular epidemiology studies of carbapenemase-producing bacteria.

The Ambler molecular classification system, based on protein homology, categorizes *β*-lactamases into four classes (A to D). The most extensive distribution class A enzyme with carbapenemase activity is KPC. The class B enzymes are metalloenzymes of the most IMP or VIM series. Besides, NDM and DIM belong to class B enzymes which are widely concerned in Asia [8]. Many OXA enzymes (OXA-23-like, OXA-48-like, class D) are considered to be responsible for the worldwide resistance epidemics as well as their detailed properties which have been extensively reported [9, 10]. The presence of *bla*_DIM-1_ has been considered lower once before, for a* Pseudomonas stutzeri *isolate from the Netherlands [11]. However, in a recent report, *bla*_DIM-1_ was found in hospital isolates belonging to the families Enterobacteriaceae, Pseudomonadaceae, Burkholderiaceae, and Comamonadaceae and forty percent of the isolates were found to contain *bla*_DIM-1_ among the tested isolates [12]. The *bla*_OXA-51_ and *bla*_OXA-23_ genes are mainly responsible for the resistance in* Acinetobacter baumannii *[13], whereas the *bla*_OXA-23-like_ genes are the most prevalent carbapenem-resistant genes identified in China [13, 14]. The *bla*_OXA-51-like_ genes have been considered exclusively chromosomally encoded, intrinsic oxacillinase genes of* Acinetobacter baumannii *and are used by many investigators for species identification and strain typing [15]. However, a number of recent reports indicate that the *bla*_OXA-51-like_ genes have been mobilized and are spreading to other* Acinetobacter* spp. by conjugative plasmids [16, 17]. While further work is required to determine carbapenem resistance of OXA-51-like group, the enzymes of this group remain a major concern, as they present the possibility that all* A. baumannii *isolates may be capable of becoming resistant to the carbapenems [18]. Therefore, in the current study, we included *bla*_KPC_, *bla*_NDM-1_, *bla*_OXA-23_, *bla*_OXA-48_, *bla*_OXA-51_, *bla*_IMP_, *bla*_VIM_, and *bla*_DIM_ in the detection panel for the DNA microarray assays.

DNA microarrays have had wide applications, including gene expression analysis [19], disease diagnosis [20], and pathogenic microorganism detection [21]. This technique is characterized by miniaturization, high-throughput, manageability, and easiness of automatization. The purpose of this work is to develop a rapid, reliable, and high-throughput DNA microarray method for detection of clinically relevant carbapenemase-encoding genes. In this assay, a reliable and portable, ultrasensitive chemiluminescence (CL) imaging DNA hybridization was developed to simultaneously detect eight genes. Plasmids were established as positive or limit of detection (LOD) reference materials. The specificity and sensitivity of the method was validated in 416 actual samples.

## 2. Materials and Methods

### 2.1. Ethics Statement

All patients provided informed consent in accordance with requirements of the Declaration of Helsinki, and the research project was approved by the Ethical Committee of Chinese People's Liberation Army (PLA) General Hospital.

### 2.2. Specimen Collection and Processing

The samples collected from PLA General Hospital of China were sputum, urine, and bacteria isolates. Sputum sample was liquefied by 4% NaOH for 30 min at room temperature with shaking and then centrifuged at 13,000 rpm for 2 min. Pellets were collected and washed. The DNA in sputum pellets and urine samples were isolated by QIAamp DNA Mini Kit according to the manufacturer's instructions. Bacteria isolates were prepared by modified boiling method. Briefly, colonies of each isolate were picked from Luria-Bertani plates and suspended in 100 *μ*L of sterilized H_2_O, followed by boiling at 95°C for 15 min and centrifugation at 12,000 rpm for 5 min. Supernatants were harvested and transferred to new tube and served as templates for PCR and microarray assays [13].

### 2.3. Primer and Probe Design

The DNA sequences of the carbapenemase (i.e., *bla*_KPC_, *bla*_NDM-1_, *bla*_OXA-23_, *bla*_OXA-48_, *bla*_OXA-51_, *bla*_IMP_, *bla*_VIM_, and *bla*_DIM_) were downloaded from GenBank (http://www.ncbi.nlm.nih.gov/genomes/). Primers and probes were designed by DNAMAN and Primer Premier. Specific primers were designed for *bla*_NDM-1_, *bla*_OXA-48_, and *bla*_DIM-1_. For *bla*_KPC_, *bla*_IMP_, *bla*_VIM_, *bla*_OXA-23-like_, and *bla*_OXA-51-like_, primers were chosen in the conserved upstream or downstream regions. Microarray probes ranging from 33 to 42 nucleotides were synthesized for these genes. These genes were amplified by multiplex PCR in separate tubes, and an internal standard probe was included for each tube for process monitoring. Finally, ten primers and seventeen probes with favorable specificities were selected (Tables 1 and 2). All the primers and probes were verified by BLAST (http://blast.ncbi.nlm.nih.gov/).

### 2.4. Construction of Reference Plasmids

Oligonucleotides of *bla*_DIM_ and *bla*_VIM_ in this study were spliced by big primer amplification method in which 40 bp fragments were concatenated into final sequences of about 280 bp. Carbapenemase-producing samples of OXA-23, OXA-48, and OXA-51 were collected from Chinese PLA General Hospital in Beijing and samples of KPC, IMP, and NDM-1 were collected from Chinese PLA Academy of Military Medical Sciences. These eight well-characterized reference strains, each carrying *bla*_KPC_, *bla*_NDM-1_, *bla*_OXA-23_, *bla*_OXA-48_, *bla*_OXA-51_, *bla*_IMP_, *bla*_VIM_, or *bla*_DIM_, were used for the design and validation of the microarray probes and primers. DNA fragments of KPC, NDM-1, OXA-23, OXA-48, OXA-51, IMP, VIM, DIM, mitochondrial DNA (mtDNA), and 16S rRNA were amplified by PCR, followed by digestion with PGM-T and cloning into DH5*α*. The cloned fragments were confirmed by sequencing the entire regions. Mitochondrial DNA and 16S rRNA were collected from cultured Hela cell and standard strains of* Escherichia coli* ATCC25922, respectively.

### 2.5. Microarray Fabrication

**Figure 1:**
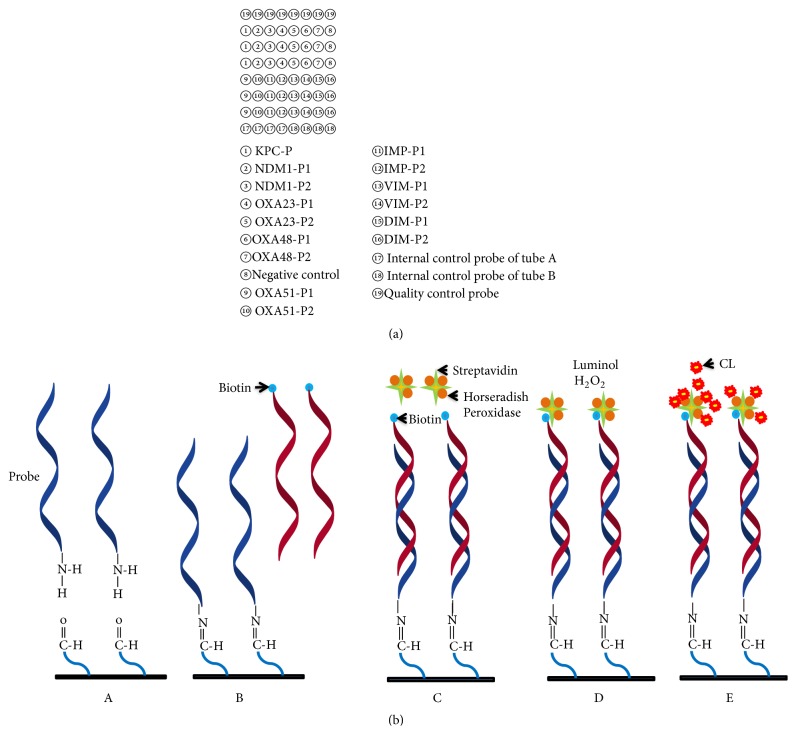
(a) The microarray layout of the target carbapenemase genes that are detected in this study. (b) Principle of hybridization and CL imaging of microarray. (A) Captured probes were fixed to the aldehyde-chip surface, (B) denatured PCR products were hybridized with capture probes, (C) horseradish peroxidase modified streptavidin was bond to biotin incorporated in hybridization, and (D) adding HRP substrates luminal and H_2_O_2_ and (E) CL signal was detected by catalyzed substrates.

All microarray probes were synthesized by the Chinese PLA Academy of Military Medical Sciences. An oligo(dT)_12_-with an amino-labeled 3′-end was conjugated to the 3′-end of all the probes, such that it served as a linker arm to be immobilized on the aldehyde modified glass surface (Baio Technology Shanghai Co., Ltd., Shanghai, China). An Oligo(dT)_ 20_ with an amino-labeled 3′-end and a biotin-labeled 5′-end served as a quality control (QC) probe. Each probe (50 *μ*M of final concentration) was spotted thrice repeatedly by using a noncontact inkjet Nano-plotter 2.1 (GeSim, Dresden, Germany) onto an aldehyde-chip after mixing with printing buffer [5% glycerol, 0.1% sodium dodecyl sulfate (SDS), 6× saline-sodium citrate buffer (SSC), and 2% (wt/vol) Ficoll 400]. Quality control (QC) probe was included to manage the standard of operation and used at 12.5 *μ*M final concentration. It was spotted eight times repeatedly in the horizontal direction to calibrate the CL signal values. Each aldehyde slide was divided into 10 blocks (11 × 11 mm) by a waterproof film to detect 10 different samples. Microarrays were placed in a dryer for 24 h at room temperature. Unbound probes were washed off by 0.2% SDS and distilled water prior to use. The layout is shown in Figure 1(a).

### 2.6. Multiplex PCR

All reverse primers for target genes and internal controls (i.e., mtDNA and 16S rRNA) were labeled by biotin at the 5′-ends for acting a CL reaction. Multiplex PCRs were performed for *bla*_KPC_, *bla*_OXA-23_, *bla*_VIM_, *bla*_DIM_, and 16S rRNA in one tube (Tube A), and *bla*_IMP_, *bla*_NDM-1_, *bla*_OXA-48_, *bla*_OXA-51_, and mtDNA in another (Tube B). PCR reaction was in 30 *μ*L and contained 15 *μ*L of 2× Multiplex PCR Mix (cwBiotech, Beijing China) and 3 *μ*L of mixed DNA templates. For Tube A, the concentrations of *bla*_OXA-23_, *bla*_VIM_, *bla*_DIM_, and 16S rRNA forward and reverse primers all were 0.1 *μ*M and 0.5 *μ*M, and for *bla*_KPC_ the forward and reverse primer were 0.2 *μ*M and 1 *μ*M, respectively. For Tube B, the concentrations of the forward and reverse primers were 0.1 *μ*M and 0.5 *μ*M, respectively. PCR was performed on a Thermal Cycler PCR system (Applied Biosystems, Foster City, CA) using the following conditions: 10 min at 95°C; 37 cycles of 30 s at 94°C, 30 s at 55°C, and 45 s at 72°C; and a final extension of 5 min at 72°C.

### 2.7. Hybridization and Signal Detection

PCR products amplified by the two multiplex PCR reactions using the same template were mixed. The mixtures were denatured at 95°C for 5min and placed on ice immediately for 5 min. Then 5 *μ*L of denatured PCR mixtures were blended with 5 *μ*L of hybridization buffer [8× SSC, 0.6% SDS, 10% formaldehyde, and 10×Denhardt]. Hybridization reaction was proceeded in a hybrid-box by incubation for 1 h at 45°C. After hybridization, slide was washed successively in 1× SSC and 0.2% SDS, 0.2× SSC, and 0.1× SSC for 30 s. Slide was air dried at room temperature. To detect the CL signals, microarray was incubated in 37°C for 30 min with 10 *μ*L of streptavidin horseradish peroxidase (Str-HRP, Sigma-Aldrich, St.Louis, USA), followed by wash with PBST (1×PBS, 0.05% Tween-20) for 30 s at room temperature. Dried microarray was covered in 10 *μ*L of premixed CL HRP substrate luminal solution and H_2_O_2_ (Millipore Corporation, Boston, USA) (Figure 1(b)). Then immediately subject to scanning by Biochip Chemiluminescence Imager, a microlight level imaging system developed in our laboratory. Signal intensities were calculated by Array Vision 7.0.

### 2.8. Identification of Carbapenem-Resistant and Susceptible Strains

The strains isolated from samples were tested using the Kirby-Bauer (K-B) method of disk diffusion according to the recommendations of the CLSI to determine their susceptibilities to imipenem (10*μ*g) and meropenem (10*μ*g).* Escherichia coli* ATCC25922 and* Pseudomonas aeruginosa* ATCC27853 were used as control strains for susceptibility testing. Isolates were considered to have a carbapenemase phenotype if they were resistant to at least one carbapenem (i.e., meropenem or imipenem) [22].

Four hundred sixteen (416) antibiotic-resistant bacterial samples were evaluated in this study. The initial samples were obtained from patients who had been hospitalized for a long time (>1 year).

### 2.9. Confirmation of the Resistance Genes by Sequencing

The resistance genes, including *bla*_KPC_, *bla*_NDM-1_, *bla*_OXA-23_, *bla*_OXA-48_, *bla*_OXA-51_, *bla*_IMP_, *bla*_VIM_, and *bla*_DIM_, that had been detected in antibiotic-resistant samples by microarray hybridization were validated by Sanger sequencing.

## 3. Results

### 3.1. Determination of Threshold Signal Intensity

To determine the threshold value for differentiating positive and negative microarray signal intensities, we have performed pilot microarray hybridization experiments using the Gram-positive bacterial strain* S. aureus* 04018 as negative controls and carbapenemase plasmids of 3×10^3^ copies/*μ*L as positive controls under the conditions specified in Materials and methods. If the signal intensity value is 10 times of the background intensity value, the probe was considered to be positive.

### 3.2. Specificity and Sensitivity of Microarray Test

**Figure 2:**
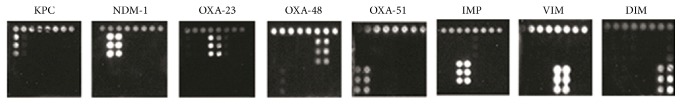
Representative microarray hybridization images with the reference carbapenemase plasmids (positive controls).

**Figure 3:**
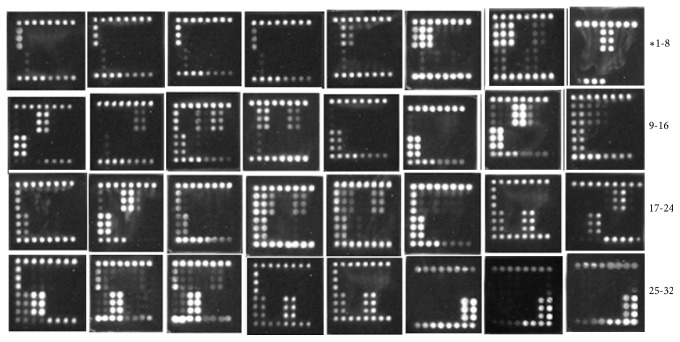
Representative images of microarray assays for clinical carbapenemase-producing specimens. *∗*, the number of clinical carbapenemase-producing specimens (Supplementary Table).

**Figure 4:**
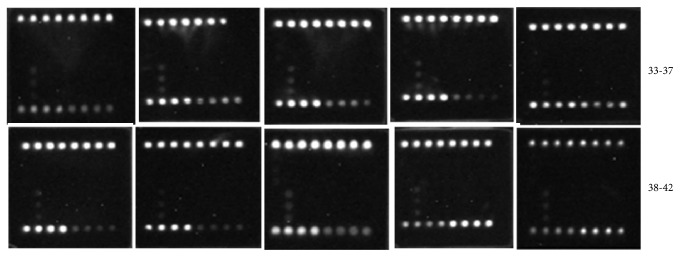
Images of microarray assays for carbapenemase-sensitive strains (negative control strains). 33,* Escherichia coli *(ATCC 25922)*; *34,* Enterococcus faecium *(ATCC 35667)*; *35,* Enterobacter cloacae *(ATCC 13047)*; *36*, Enterococcus faecalis *(ATCC 29212)*; *37*, Pseudomonas aeruginosa *(ATCC 27853)*;* 38,* Acinetobacter baumannii *(ATCC 19606)*; *39,* Staphylococcus aureus *(ATCC25923)*; *40,* Streptococcus pneumoniae *(ATCC 49619*); *41,* Klebsiella pneumonia *(ATCC700603*); *42*, Streptococcus pneumonia *(ATCC 9007(serotype C)*).* ATCC, American Type Culture Collection.

To evaluate the specificity of the microarray method, we performed microarray hybridization assays for the reference carbapenemase plasmids (i.e., positive controls) (Figure 2), clinical carbapenem-resistant samples (Figure 3), and ten negative controls that were from ATCC standard strains and were sensitive to carbapenem (Figure 4). As shown by the microarray images, our method could effectively distinguish between the carbapenem resistance and carbapenem-sensitive genotypes among the clinical bacterial specimens with high specificity.

**Figure 5:**
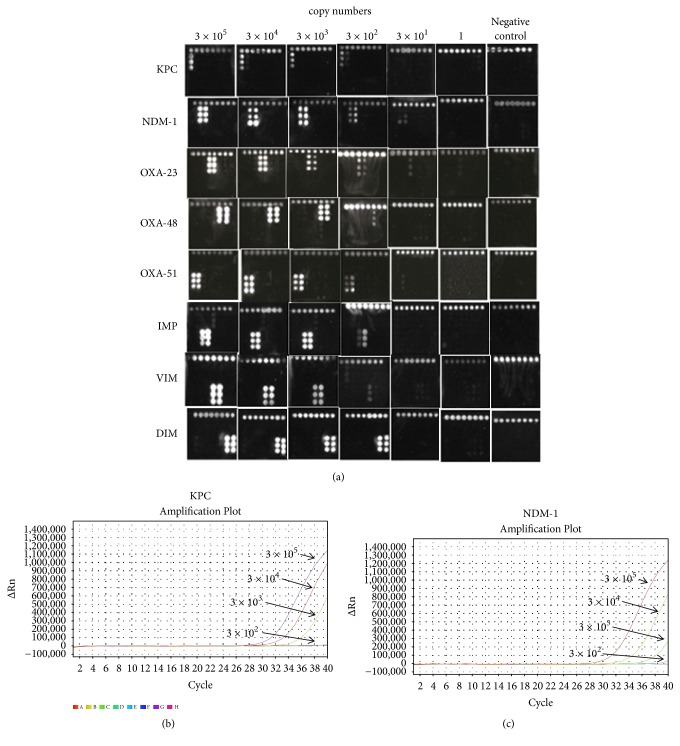
(a) Sensitivity of the microarray assays. The indicated carbapenemase reference plasmids were subject to series dilutions and hybridized to the DNA microarrays. The plasmids copy numbers are diluted from 3×10^1^ to 3×10^5^ copies/*μ*L. (b) KPC reference plasmids (3×10^1^ to 3×10^5^ copies/*μ*L) were detected by real-time PCR, (c) NDM-1 reference plasmids (3×10^1^ to 3×10^5^ copies/*μ*L) were detected by real-time PCR.

To evaluate the sensitivity of the microarray assay, we diluted the reference carbapenemase plasmids into various concentrations (i.e., from 3×10^1^ copies/*μ*L to 3×10^5^ copies/*μ*L). The different copy numbers of DNA were hybridized to the microarrays. The detection images were shown in Figure 5(a). In general, the microarrays yielded satisfactory sensitivity. For most reference plasmids, the detection limit was as low as 30 copies/*μ*L (Figure 5(a)). Diagnostic Kit for Bacterial Resistance Gene KPC (PCR-Fluorescence Probing) and Diagnostic Kit for Bacterial Resistance Gene NDM-1 (PCR-Fluorescence Probing) (Puruikang Biotech, Shenzhen, China) were also used to detect KPC and NDM-1 reference plasmids (3×10^1^ to 3×10^5^ copies/*μ*L), respectively. Real-time PCR amplified by ABI Prism 7500 real-time PCR apparatus (Applied Biosystems, Foster City, US) and the results of sensitivity comparison between microarray assay and Real-time PCR showed they had similar sensitivities (Figures 5(b) and 5(c)), indicating that our DNA microarrays could be applied to the clinical detection of the carbapenemase-producing samples.

### 3.3. Stability of Microarray Assay

Diluted carbapenemase plasmids (3×10^5^ copies/*μ*L) and negative controls* S. aureus* 04018 were used to evaluate how the microarray assays performed as far as repeatability is concerned. The 3×10^3^copy/*μ*L plasmid of each target gene was selected as the template to detect for the determination of interchip and intrachip variation, and a negative control was set up without template. The experiment was repeated three times, and the repeatability of interchip and intrachip variation were evaluated. Coefficient of variation (CV) = SD/mean of signal intensities×100%.

Statistical analysis showed that, for all target gene probes, the intrachip and interchip CV values of 8 probes ranged from 3.58% to 11.02% (below 15%), suggesting a favorable repeatability of the DNA microarray detection method (Table 3).

### 3.4. Phenotypic Resistance

About 78% clinical specimens (326/416) were resistant to both imipenem and meropenem, 77 samples were susceptible to both imipenem and meropenem, three samples were resistant to imipenem but sensitive to meropenem, and five samples were reversely sensitive to imipenem but resistant to meropenem as well as five samples were intermediary to imipenem but sensitive to meropenem. Most carbapenem-resistant samples were nonsusceptible to diverse antibiotics containing cephalosporins, fluoroquinolone, aminoglycosides, etc. The rates of nonsusceptibility to different antimicrobial agents were commonly >90%. Clinical information and phenotypic results of all clinical samples were shown in Supplementary Table (available here).

### 3.5. Detection of Carbapenemase-Producing Strains by Microarray in Clinical Samples

A total of 416 clinical samples collected from Chinese PLA General Hospital were tested. The majority of the samples had previously been well-characterized as carbapenem resistance using K-B method. The microarrays for these bacteria revealed that 78% (325) of the samples carried one or more carbapenemase genes and that in some samples more than one* bla* gene had been identified (i.e., 496 genes were found in 325 specimens). The genotyping results of the clinical samples are listed as follows: 256 (62%) carried *bla*_KPC_, 137 (33%) carried *bla*_OXA-51_, 40 (9.6%) carried *bla*_OXA-23_, 22 (5.3%) carried *bla*_OXA-48_, 27 (6.5%) carried *bla*_NDM-1_, 5 (1.2%) carried *bla*_IMP_, 3 (0.7%) carried *bla*_VIM_, and 6 (1.4%) carried *bla*_DIM_ (Table 4). Therefore, *bla*_KPC_ was the most frequent carbapenemase gene found in the antibiotic-resistant sample. Most interestingly, we found that *bla*_OXA-51_ frequently coexisted with other* bla* genes. For example, 89 samples that carried *bla*_KPC_ also carried *bla*_OXA-51_; all 40 samples of *bla*_OXA-23_ carried *bla*_OXA-51_; 9 samples carried all three* bla* genes: *bla*_KPC_, *bla*_OXA-51_, and *bla*_OXA-23_; and 5 samples carrying *bla*_OXA-48_ also carried both *bla*_OXA-51_ and *bla*_KPC_. It is worth noting that all carbapenemase genes in the 325 antibiotic-resistant samples had been verified by sequencing. In parallel, we also amplified the isolates that were susceptible to carbapenems performed in phenotypic tests as well as recruited in our microarray assays. The overall concordance between the microarray-based assay and the reference methods (standard DNA sequencing) was 97.8%, suggesting that our microarray is a highly reliable method for detecting the carbapenemase-producing GNB in the clinic.

Fourteen samples which failed to detect any genes by microarray but resistant to either imipenem or meropenem in phenotypic test could not be amplified, determining 96.6% concordance between the phenotypic and microarray tests. Taken together, the newly developed microarray detection method is comparable to the conventional antibiotic susceptibility test and therefore may be suitable for clinical applications.

## 4. Discussion

In this study, we developed a novel microarray method to detect carbapenemase genes that could be applied to clinical diagnosis and identification of carbapenemase-producing GNB. We included eight carbapenemase genes that have been shown or potential display to be most clinically relevant, *bla*_KPC_, *bla*_NDM-1_, *bla*_OXA-23_, *bla*_OXA-48_, *bla*_OXA-51_, *bla*_IMP_, *bla*_VIM_, and *bla*_DIM_, in the design of the microarray chips. MtDNA and 16S rRNA were chosen as internal controls because our samples were taken from human and the detected target genes were from bacteria. The high copy number sequence of mtDNA was used to monitor and control all PCR reactions and hybrids operations, and 16S rRNA was used to prove that the DNA microarray system could detect bacterial genes from all samples. The specificity, sensitivity, and reproducibility of the proposed method were highly favorable for clinical applications. Most importantly, the microarray results of the 416 clinical samples showed highly consistent agreement with results obtained from direct sequencing or antibiotic susceptibility tests.

It was intriguing for us to identify a number of resistant strains coharboring two or more carbapenemase genes; for instance, *bla*_KPC_ frequently coexisted with other genes and *bla*_OXA-51_ always coexisted with *bla*_OXA-23_ in* Acinetobacter bau mannii*, indicating a more serious threat than before, when it came to the control and management of the extremely drug-resistant bacterial infections [23]. Of the transferable molecular class B metallo-*β*-lactamases, IMP, VIM, and NDM were common, while DIM was endemic [10]. However, in our study, *bla*_IMP_ and *bla*_VIM_ were detected in 5 and 3 samples, respectively, yet *bla*_DIM_ was in 6 specimens, which indicated that it might be necessary to improve the attention for DIM in the later study (Table 4 and Supplementary Table).

The DNA microarray could detect bacterial carbapenemase genes from not only clinical sputum, urine samples, and colony or bacterial culture in this study, but also specimens of pleural effusion, cerebrospinal fluid, oral swab, and throat swab even environmental swab (data not shown) directly. This remarkable capability of compatibility to detect of several original samples made it faster to obtain result than similar microarrays Check-MDR CT102 [24, 25] and VITEK2 [26] that should detect cultured bacterial isolates. The microarray was rapid and portable, when starting from the clinical sample, less than 7 hours with overall 2 hours of hands-on time, enabling one day analysis. Whole detection operation of the DNA microarray consisted of 5 steps and costs 4-5 h including PCR amplification, and whole detection did not need sophisticated instrument, which was far simpler than Check-MDR CT102. The DNA microarray could detect 9 specimens at one chip one time and the cost per sample was below five dollars, which was far cheaper than Check-MDR CT102 and VITEK2. In microarray assay, a proprietary CL imaging system was developed in our laboratory. The Biochip Chemiluminescence Imager relied on charge-coupled device (CCD) camera imaging technology and equipped with a power supply unit for portable use. The new CL imager had a lower cost ($3000) than other commercial CCD imaging technology CL imagers (e.g., Amersham Imager 600, GE Healthcare Life Sciences) and much faster than other visual microarray system which was based on quantum dot-catalyzed silver deposition. The newly designed DNA microarray system had yielded high specificity, sensitivity, and reproducibility in detecting the eight carbapenemase genes among the clinical specimens. For *bla*_NDM-1_, *bla*_OXA-23_, *bla*_OXA-48_, *bla*_OXA-51_, *bla*_IMP_, *bla*_VIM_, and *bla*_DIM_, there were two different probes (22-30 nt) designed to hybridize, respectively, which could effectively minimize the false hybridization signals. Our study has demonstrated that the microarrays with greatly simplified the protocol to determine the cabapenemase-encoding genes in the clinical samples and can offer an efficient means for the molecular epidemiological studies of cabapenemase genes: the emergence of the resistant genes may potentially be traced back to their origins where community- and hospital-based bacterial infections were frequently happening. On the other hand, microarray hybridization is highly sensitive. In this study, they showed similar sensitivities as the real-time PCR kit. We could detect as low as 30 copies/*μ*L of DNA targets. Furthermore, the DNA microarrays can generally detect target DNAs with much larger dynamic ranges. Finally, the microarray method is also highly reproducible: we have shown that the averaged coefficient of variations (CV %) for interchip and intrachip experiments were low, and most of them were less than 10%. Therefore, we propose that the new microarray method has a great potential to be applied to clinical studies.

The microarray based on multi-PCR, which made it possible to detect multiple resistant genes at the same time in a single tube. But it could not effectively identify a variety of fragments by electrophoretic separation. Direct sequencing of DNA is not suitable for identification of multiple PCR products. The microarray is used to confirm the results of multiple PCR. The concordance of microarray to PCR of detection 8 carbapenemase genes ranges from 95.7%-100% (Table 4).

The microarray had some limitations, because resistance to carbapenems in particular was produced through several mechanisms: such as synthesis of carbapenem *β*-lactamases, efflux pumps [27], loss of membrane permeability [28], and penicillin-binding proteins variants. The DNA microarray could not cover genes form all the mechanisms. Fourteen samples in our study which failed to detect any genes by microarray but resistant to either imipenem or meropenem in phenotypic test could not be amplified, indicating resistant genes from other mechanisms were beyond of detection reach of the microarray. Whole genome sequencing (WGS) has potential to detect many different molecular mechanisms leading to resistance. Our lab has taken WGS as new powerful technology to find novel resistant genes located in bacterial plasmid or genome DNA, as well as new resistant mechanisms. However, WGS-based antimicrobial susceptibility testing in clinical laboratories remain the current high-cost and taking more time [29]. In clinical testing, using PCR-based microarray to detect some specific resistance genes simultaneously was more economical and faster. In this study, multi-PCR amplification was divided into two tubes, and reducing PCR amplification systems (amplifying 8 carbapenemase genes in one tube) might increase sensitivity [30].

In conclusion, we developed a new microarray detection system that could directly detect eight carbapenemase genes from several kinds of clinical specimens. It was convenient, readily to be customized for high-throughput detection, and could be easily adapted for clinical applications.

## Figures and Tables

**Table 1:** The primer sequences for microarray.

Primer ^a^	Sequence (5′-3′)	Positions ^b^	Targeted gene/ GenBank accession
KPC-F	CTGGGCAGTCGGAGACAAAA	681-700	KPC/ KX619622.1
KPC-R	AGACGGCCAACACAATAGGT	765-784	
NDM1-F	GAATGTCTGGCAGCACACT	168-186	NDM-1/ KX249707
NDM1-R	TGGCATAAGTCGCAATCC	407-424	
OXA23-F	GCAGTCCCAGTCTATCAGGA	379-398	OXA-23/NG_049726
OXA23-R	CCCAACCAGTCTTTCCAA	641-658	
OXA48-F	TCGGGCAATGTAGACAGTT	548-556	OXA-48/ NG_049762
OXA48-R	CACCAGCCAATCTTAGGTTC	746-765	
OXA51-F	GCTCGTCGTATTGGACTTGA	406-425	OXA-51/ KX609247
OXA51-R	TGTGCCTCTTGCTGAGG	523-539	
IMP-F	GTAATTGACACTCCATTTAC	291-309	IMP /NG_049172
IMP-R	GCGGACTTTGGCCAAGCTTC	674-693	
VIM-F	TGGTGAGTATCCGACAG	190-206	VIM/NG_050336
VIM-R	ATGAAAGTGCGTGGAG	433-448	
DIM-F	GCTTGTCTTCGCTTGCTAA	38-56	DIM/NG_049077
DIM-R	ATTCCTGCGGTTCTATCCT	293-311	
mtDNA-F	GTCGAAGGTGGATTTAGCAGTAA	1413-1435	mtDNA/MG182040
mtDNA-R	GTAAGGTGGAGTGGGTTTGGG	1684-1704	
182-F	AGAGTTTGATCMTGGCTCAG	1-20	16S rRNA/ LN612729
756-R	CGTATTACCGCGGCTGCTG	518-530	

^a^ F, forward primer; R, reverse primer; all reverse primers have biotin conjugated at 5′-ends.

^b^ Positions refer to the nucleotide numberings of the corresponding GenBank genes.

**Table 2:** The probe sequences for microarray.

Probe	Sequences (5′-3′) ^a^	Targeted gene
KPC-P	CAAATGACTATGCCGTCGTCTGGCC	KPC
NDM1-P1	ACCGATGACCAGACCGCCCAGATCCTCAAC	NDM-1
NDM1-P2	TCAGGACAAGATGGGCGGTATGGAC	NDM-1
OXA23-P1	TTTTAGAAGAGAGTAATGGCTACAAAA	OXA-23
OXA23-P2	ATTGGACAGCAGGTTGATAATTTCTGG	OXA-23
OXA48-P1	CGAATTTCGGCCACGGAGCAAATCAGCTT	OXA-48
OXA48-P2	CAGCGTATTGTCAAACAAGCCATGC	OXA-48
OXA51-P1	GAAGTGAAGCGTGTTGGTTATG	OXA-51
OXA51-P2	ATATCGGTACCCAAGTCGATAATTTTTGGC	OXA-51
IMP-P1	GGCTAGTTAAAAATAAAATTGAAG	IMP
IMP-P2	CCCACGTATGCRTCTGAATTAAC	IMP
VIM-P1	TGGTGTTTGGTCGCATATCGCAACG	VIM
VIM-P2	CTCATTGTCCGTGATGGTGATGAG	VIM
DIM-P1	GTCAGTTCAAACGGCCTTGTTGTCATAGATT	DIM
DIM-P2	CTTGGTCAGACCGAGATACAGAAACGCTCG	DIM
mtDNA-P	ATGTCCTTTGAAGTATACTTGAGGAGTT	mitochondria
551-P	ACTCCTACGGGAGGCAGCAGTT	16S rRNA
Quality control ^b^	TTTTTTTTTTTTTTTTTTTT	Oligo dT_20_

^a^ An oligonucleotide of 12 T's with an amino-labeled 3′-end was conjugated to the 3′-ends of all probes. ^b^ An oligonucleotide of 20 T's with an amino-labeled 3′-end, biotin-labeled 5′-end was used as microarray quality control.

**Table 3:** Statistics of the microarray repeatability.

	Repeatability (CV%)			
	Intra-chip experiments			Inter-chip experiment
Repeat times (n)	1	2	3	3

KPC	5.23	4.75	9.85	10.16
NDM-1	3.67	4.12	6.44	6.71
OXA-23	4.47	5.03	7.82	7.54
OXA-48	3.58	3.87	6.83	6.42
OXA-51	4.45	5.31	8.09	7.88
IMP	6.84	6.95	10.24	11.02
VIM	5.87	6.04	9.97	10.19
DIM	4.02	3.89	6.52	6.67

**Table 4:** Microarray results on various clinical samples harboring carbapenemase genes.

Species		*P. aeruginosa*	*A. baumann*	*E. coli*	*K. pneumoniae*	*S. marcescens*	*S. maltophilia*	*Flavobacterium meningosepticum*	*R. mannitolilytica*	*Achromobacter xylosoxidans*	*Burkholderia cepacia*	*Klebsiella oxytoca*	*Enterobacter cloacae*	*E. aerogenes*	Total
No^a^. of samples		263	42	36	55	5	4	3	1	1	1	2	2	1	416

No. with *bla*_KPC_ gene	PCR^b^	193	15	7	38	0	2	3	0	1	1	0	0	0	260
	Array^c^	190	15	7	37	0	2	3	0	1	1	0	0	0	256
	% Concordance of *bla*_KPC_ results	98.4	100	100	97.4	100	100	100	100	100	100	100	100	100	98.5^d^

No. with *bla*_NDM-1_ gene	PCR	19	2	0	5	0	1	0	0	0	0	0	0	0	27
	Array	19	2	0	5	0	1	0	0	0	0	0	0	0	27
	% Concordance of *bla*_NDM-1_ results	100	100	100	100	100	100	100	100	100	100	100	100	100	100^e^

No. with *bla*_OXA-23_ gene	PCR	8	31	1	0	0	1	0	0	0	0	0	0	0	41
	Array	8	30	1	0	0	1	0	0	0	0	0	0	0	40
	% Concordance of *bla*_OXA-23_ results	100	96.8	100	100	100	100	100	100	100	100	100	100	100	97.6^f^

No. with *bla*_OXA-48_ gene	PCR	20	1	1	1	0	0	0	0	0	0	0	0	0	23
	Array	19	1	1	1	0	0	0	0	0	0	0	0	0	22
	% Concordance of *bla*_OXA-48_ results	95	100	100	100	100	100	100	100	100	100	100	100	100	95.7^g^

No. with *bla*_OXA-51_ gene	PCR	84	40	5	7	0	2	1	0	1	0	0	0	0	140
	Array	82	39	5	7	0	2	1	0	1	0	0	0	0	137
	% Concordance of *bla*_OXA-51_ results	97.6	100	100	100	100	100	100	100	100	100	100	100	100	97.9^h^

No. with *bla*_IMP_ gene	PCR	4	1	0	0	0	0	0	0	0	0	0	0	0	5
	Array	4	1	0	0	0	0	0	0	0	0	0	0	0	5
	% Concordance of *bla*_IMP_ results	100	100	100	100	100	100	100	100	100	100	100	100	100	100^i^

No. with *bla*_VIM_ gene	PCR	3	0	0	0	0	0	0	0	0	0	0	0	0	3
	Array	3	0	0	0	0	0	0	0	0	0	0	0	0	3
	% Concordance of *bla*_VIM_ results	100	100	100	100	100	100	100	100	100	100	100	100	100	100^j^

No. with *bla*_DIM_ gene	PCR	4	1	0	0	0	0	0	1	0	0	0	0	0	6
	Array	4	1	0	0	0	0	0	1	0	0	0	0	0	6
	% Concordance of *bla*_DIM_ results	100	100	100	100	100	100	100	100	100	100	100	100	100	100^k^

Total no. of strains that agree/total no. of strains		257/263	38/40	36/36	54/55	5/5	4/4	3/3	1/1	1/1	1/1	2/2	2/2	1/1	407/416

% agreement for all strains		97.7	95	100	98.2	100	100	100	100	100	100	100	100	100	97.8

^a^ All tested samples include sputum, urine, and cultured isolates; the specific clinical information and test results of each sample are shown in the Supplementary Table.

^b^ Results obtained with classical PCR/sequencing.

^c^ Microarray results were obtained using carbapenemase-array designed in this study.

^d^ A sensitivity of 98.5%, specificity of 100%, positive predictive value of 100%, and negative predictive value of 97.5% for KPC detection.

^e^ A sensitivity of 100%, specificity of 100%, positive predictive value of 100%, and negative predictive value of 100%f or NDM-1 detection.

^f^ A sensitivity of 97.6%, specificity of 100%, positive predictive value of 100%, and negative predictive value of 99.7% for OXA-23 detection.

^g^ A sensitivity of 95.7%, specificity of 100%, positive predictive value of 100%, and negative predictive value of 99.7% for OXA-48 detection.

^h^ A sensitivity of 97.9%, specificity of 100%, positive predictive value of 100%, and negative predictive value of 98.9% for OXA-51 detection.

^I^ A sensitivity of 100%, specificity of 100%, positive predictive value of 100%, and negative predictive value of 100% for IMP detection.

^j^ A sensitivity of 100%, specificity of 100%, positive predictive value of 100%, and negative predictive value of 100% for VIM detection.

^k^ A sensitivity of 100%, specificity of 100%, positive predictive value of 100%, and negative predictive value of 100% for DIM detection.

## Data Availability

The date generated and analyzed during this study are available from the corresponding author on reasonable request.

## Supplementary Materials

Supplementary Materials Additional file 1: Supplementary Table. Clinical information and detection results of carbapenemase-producing samples.
