# Supplementary material for: Laboratory and Clinical Evaluation of DNA Microarray for the Detection of Carbapenemase Genes in Gram-Negative Bacteria from Hospitalized Patients
Source: Biomed Res Int. 2019 May 13;2019:8219748. doi: 10.1155/2019/8219748 (PMC6535891; doi:10.1155/2019/8219748)
Supplement: Supplementary Materials — Additional file 1: Supplementary Table. Clinical information and detection results of carbapenemase-producing samples. [file 8219748.f1.docx]

| Supplementary Table Detection results of clinical carbapenemase-producing strains | | | | | | | | | | | | | | | |
| --- | --- | --- | --- | --- | --- | --- | --- | --- | --- | --- | --- | --- | --- | --- | --- |
| ID | gender | age | sample type | clinical diagnosis | species | imipenem | meropenem | KPC | NDM  -1 | OXA  -23 | OXA  -48 | OXA  -51 | IMP | VIM | DIM |
| 1 | male | 88 | sputum | pulmonary infection | *P. aeruginosa* | R | R | **+** | **-** | **-** | **-** | **-** | **-** | **-** | **-** |
| 2 | male | 91 | sputum | pulmonary infection | *P. aeruginosa* | R | R | **+** | **-** | **-** | **-** | **-** | **-** | **-** | **-** |
| 3 | male | 100 | sputum | bilateral pneumonia | *P. aeruginosa* | R | R | **+** | **-** | **-** | **-** | **-** | **-** | **-** | **-** |
| 4 | male | 93 | sputum | pneumonia | *P. aeruginosa* | R | R | **+** | **-** | **-** | **-** | **-** | **-** | **-** | **-** |
| 5 | male | 86 | sputum | pneumonia, cerebral thrombosis | *P. aeruginosa* | R | R | **-** | **+** | **-** | **-** | **-** | **-** | **-** | **-** |
| 6 | male | 92 | sputum | bilateral pneumonia | *P. aeruginosa* | R | R | **+** | **+** | **-** | **-** | **-** | **-** | **-** | **-** |
| 7 | male | 89 | sputum | pneumonia | *P. aeruginosa* | R | R | **+** | **+** | **-** | **-** | **-** | **-** | **-** | **-** |
| 8 | male | 97 | sputum | pneumonia | *P. aeruginosa* | R | R | **-** | **-** | **+** | **-** | **+** | **-** | **-** | **-** |
| 9 | male | 97 | sputum | bilateral pneumonia | *P. aeruginosa* | R | R | **-** | **-** | **+** | **-** | **+** | **-** | **-** | **-** |
| 10 | male | 102 | sputum | pneumonia | *P. aeruginosa* | R | R | **-** | **-** | **-** | **+** | **-** | **-** | **-** | **-** |
| 11 | male | 90 | sputum | bilateral pneumonia | *P. aeruginosa* | R | R | **+** | **-** | **-** | **+** | **+** | **-** | **-** | **-** |
| 12 | male | 98 | sputum | pulmonary infection | *P. aeruginosa* | R | R | **-** | **+** | **-** | **+** | **-** | **-** | **-** | **-** |
| 13 | male | 96 | sputum | chronic bronchitis | *P. aeruginosa* | R | R | **-** | **-** | **-** | **-** | **+** | **-** | **-** | **-** |
| 14 | male | 90 | sputum | pulmonary infection, Parkinson's disease | *P. aeruginosa* | R | R | **-** | **-** | **-** | **-** | **+** | **-** | **-** | **-** |
| 15 | male | 92 | sputum | bilateral pneumonia, multi-Cerebral Infarction | *A. baumannii* | R | R | **-** | **-** | **+** | **-** | **+** | **-** | **-** | **-** |
| 16 | female | 98 | sputum | chronic myelomonocytic leukemia | *K. pneumoniae* | R | R | **+** | **+** | **-** | **-** | **+** | **-** | **-** | **-** |
| 17 | female | 98 | sputum | chronic myelomonocytic leukemia | *K. pneumoniae* | R | R | **+** | **-** | **-** | **-** | **+** | **-** | **-** | **-** |
| 18 | male | 88 | sputum | bilateral pneumonia | *P. aeruginosa* | R | R | **-** | **-** | **+** | **-** | **+** | **-** | **-** | **-** |
| 19 | male | 90 | sputum | radiation enteritis | *P. aeruginosa* | R | R | **+** | **-** | **-** | **-** | **+** | **-** | **-** | **-** |
| 20 | male | 86 | sputum | pneumonia, hydrocephalus | *P. aeruginosa* | R | R | **+** | **+** | **-** | **+** | **+** | **-** | **-** | **-** |
| 21 | male | 86 | sputum | bilateral pneumonia | *K. pneumoniae* | R | R | **+** | **+** | **-** | **+** | **+** | **-** | **-** | **-** |
| 22 | male | 59 | sputum | bilateral pneumonia | *Flavobacterium meningosepticum* | R | I | **+** | **-** | **-** | **-** | **+** | **-** | **-** | **-** |
| 23 | female | 92 | sputum | pneumonia | *P. aeruginosa* | R | R | **+** | **+** | **-** | **+** | **+** | **-** | **-** | **-** |
| 24 | male | 94 | sputum | colorectal cancer, pneumonia | *P. aeruginosa* | R | R | **-** | **-** | **-** | **+** | **-** | **+** | **-** | **-** |
| 25 | male | 93 | sputum | pneumonia | *P. aeruginosa* | R | R | **+** | **-** | **-** | **-** | **+** | **+** | **-** | **-** |
| 26 | male | 96 | sputum | pneumonia | *P. aeruginosa* | R | R | **+** | **-** | **-** | **-** | **-** | **+** | **-** | **-** |
| 27 | male | 92 | sputum | bilateral pneumonia | *P. aeruginosa* | R | R | **+** | **-** | **-** | **-** | **-** | **+** | **-** | **-** |
| 28 | male | 86 | sputum | advanced lung cancer | *P. aeruginosa* | R | R | **+** | **-** | **-** | **-** | **+** | **-** | **+** | **-** |
| 29 | male | 92 | sputum | bilateral pneumonia | *P. aeruginosa* | R | R | **+** | **-** | **-** | **-** | **+** | **-** | **+** | **-** |
| 30 | male | 86 | sputum | bilateral pneumonia | *P. aeruginosa* | R | R | **-** | **-** | **-** | **-** | **-** | **-** | **-** | **+** |
| 31 | male | 90 | sputum | pneumonia | *A. baumannii* | R | R | **-** | **-** | **-** | **-** | **-** | **-** | **-** | **+** |
| 32 | male | 92 | sputum | bilateral pneumonia | *Ralstonia mannitolilytica* | R | R | **-** | **-** | **-** | **-** | **-** | **-** | **-** | **+** |
| 33 | male | 95 | sputum | bilateral pneumonia | *P. aeruginosa* | R | R | **+** | **-** | **-** | **+** | **+** | **-** | **-** | **-** |
| 34 | male | 87 | sputum | bilateral pneumonia | *P. aeruginosa* | S | S | **-** | **-** | **-** | **-** | **-** | **-** | **-** | **-** |
| 35 | male | 92 | sputum | pneumonia | *P. aeruginosa* | S | S | **-** | **-** | **-** | **-** | **-** | **-** | **-** | **-** |
| 36 | male | 91 | sputum | severe pneumonia | *Stenotrophomonas maltophilia* | R | R | **+** | **-** | **-** | **-** | **+** | **-** | **-** | **-** |
| 37 | male | 90 | sputum | bilateral pneumonia | *K. pneumoniae* | S | S | **-** | **-** | **-** | **-** | **-** | **-** | **-** | **-** |
| 38 | male | 81 | sputum | bilateral pneumonia | *K. pneumoniae* | R | R | **-** | **+** | **-** | **-** | **-** | **-** | **-** | **-** |
| 39 | male | 91 | sputum | bilateral pneumonia | *A. baumannii* | R | R | **+** | **-** | **-** | **-** | **+** | **-** | **-** | **-** |
| 40 | male | 100 | sputum | bilateral pneumonia | *P. aeruginosa* | R | R | **+** | **-** | **-** | **-** | **-** | **-** | **+** | **-** |
| 41 | male | 82 | sputum | pneumonia | *E. coli* | R | R | **+** | **-** | **-** | **-** | **-** | **-** | **-** | **-** |
| 42 | male | 95 | sputum | pneumonia | *Stenotrophomonas maltophilia* | R | R | **-** | **+** | **-** | **-** | **-** | **-** | **-** | **-** |
| 43 | male | 73 | sputum | advanced lung cancer | *A. baumannii* | R | R | **-** | **-** | **+** | **-** | **+** | **-** | **-** | **-** |
| 44 | male | 82 | sputum | pneumonia | *Stenotrophomonas maltophilia* | R | R | **-** | **-** | **+** | **-** | **+** | **-** | **-** | **-** |
| 45 | male | 86 | sputum | chronic obstructive pulmonary disease (COPD) | *Stenotrophomonas maltophilia* | R | R | **+** | **-** | **-** | **-** | **-** | **-** | **-** | **-** |
| 46 | male | 80 | sputum | bilateral pneumonia | *P. aeruginosa* | R | R | **+** | **-** | **-** | **-** | **-** | **-** | **-** | **-** |
| 47 | male | 89 | sputum | bilateral pneumonia | *P. aeruginosa* | 17 | R | **+** | **-** | **-** | **-** | **-** | **-** | **-** | **-** |
| 48 | male | 87 | sputum | bilateral pneumonia | *K. pneumoniae* | S | S | **-** | **-** | **-** | **-** | **-** | **-** | **-** | **-** |
| 49 | female | 90 | sputum | bilateral pneumonia | *E. coli* | S | S | **-** | **-** | **-** | **-** | **-** | **-** | **-** | **-** |
| 50 | male | 88 | sputum | bilateral pneumonia | *K. pneumoniae* | R | R | **+** | **-** | **-** | **-** | **-** | **-** | **-** | **-** |
| 51 | male | 94 | sputum | COPD | *E. coli* | S | S | **-** | **-** | **-** | **-** | **-** | **-** | **-** | **-** |
| 52 | male | 84 | sputum | bilateral pneumonia | *E. coli* | S | S | **-** | **-** | **-** | **-** | **-** | **-** | **-** | **-** |
| 53 | male | 85 | sputum | bilateral pneumonia | *P. aeruginosa* | R | R | **-** | **+** | **-** | **-** | **-** | **-** | **-** | **-** |
| 54 | male | 92 | sputum | bilateral pneumonia | *A. baumannii* | R | R | **+** | **-** | **-** | **-** | **+** | **-** | **-** | **-** |
| 55 | male | 88 | sputum | bilateral pneumonia | *A. baumannii* | R | R | **+** | **-** | **-** | **-** | **+** | **-** | **-** | **-** |
| 56 | female | 95 | sputum | bilateral pneumonia | *P. aeruginosa* | I | S | **+** | **-** | **-** | **-** | **-** | **-** | **-** | **-** |
| 57 | male | 86 | sputum | bilateral pneumonia | *P. aeruginosa* | S | S | **-** | **-** | **-** | **-** | **-** | **-** | **-** | **-** |
| 58 | male | 86 | sputum | advanced lung cancer | *P. aeruginosa* | R | R | **+** | **-** | **-** | **-** | **-** | **-** | **-** | **-** |
| 59 | male | 92 | sputum | bilateral pneumonia | *P. aeruginosa* | R | R | **-** | **+** | **-** | **-** | **-** | **-** | **-** | **-** |
| 60 | male | 89 | sputum | pneumonia | *P. aeruginosa* | R | R | **+** | **-** | **-** | **-** | **-** | **-** | **-** | **-** |
| 61 | male | 97 | sputum | pneumonia | *P. aeruginosa* | R | R | **+** | **-** | **-** | **-** | **-** | **-** | **-** | **-** |
| 62 | male | 97 | sputum | bilateral pneumonia | *P. aeruginosa* | R | R | **+** | **-** | **-** | **-** | **-** | **-** | **-** | **-** |
| 63 | male | 102 | sputum | pneumonia | *P. aeruginosa* | R | I | **+** | **-** | **-** | **-** | **-** | **-** | **-** | **-** |
| 64 | male | 90 | sputum | radiation enteritis | *P. aeruginosa* | R | R | **-** | **-** | **-** | **-** | **-** | **-** | **-** | **-** |
| 65 | male | 94 | sputum | cancer colon | *P. aeruginosa* | R | R | **+** | **-** | **-** | **-** | **-** | **-** | **-** | **-** |
| 66 | male | 92 | sputum | cerebral infarction | *P. aeruginosa* | R | R | **+** | **-** | **-** | **-** | **-** | **-** | **-** | **-** |
| 67 | male | 89 | sputum | bilateral pneumonia | *P. aeruginosa* | R | R | **+** | **-** | **-** | **-** | **-** | **-** | **-** | **-** |
| 68 | male | 98 | sputum | pulmonary infection | *P. aeruginosa* | R | R | **+** | **-** | **+** | **-** | **+** | **-** | **-** | **-** |
| 69 | male | 96 | sputum | coronary artery disease, chronic bronchitis | *P. aeruginosa* | R | R | **+** | **-** | **+** | **-** | **+** | **-** | **-** | **-** |
| 70 | male | 90 | sputum | Parkinson’s disease | *P. aeruginosa* | R | R | **+** | **-** | **+** | **-** | **+** | **-** | **-** | **-** |
| 71 | male | 92 | sputum | cerebral infarction | *A. baumannii* | R | R | **-** | **-** | **+** | **-** | **+** | **-** | **-** | **-** |
| 72 | male | 93 | sputum | hypertension | *K. pneumoniae* | S | S | **-** | **-** | **-** | **-** | **-** | **-** | **-** | **-** |
| 73 | female | 98 | urine | urinary infection | *K. pneumoniae* | R | R | **+** | **-** | **-** | **-** | **-** | **-** | **-** | **-** |
| 74 | female | 98 | sputum | chronic myelomonocytic leukaemia | *K. pneumoniae* | R | R | **+** | **-** | **-** | **-** | **-** | **-** | **-** | **-** |
| 75 | male | 92 | sputum | bilateral pneumonia | *Burkholderia cepacia* | R | R | **+** | **-** | **-** | **-** | **-** | **-** | **-** | **-** |
| 76 | male | 85 | sputum | fracture | *Enterobacter cloacae* | S | S | **-** | **-** | **-** | **-** | **-** | **-** | **-** | **-** |
| 77 | male | 80 | sputum | cancer colon | *K. pneumoniae* | S | S | **-** | **-** | **-** | **-** | **-** | **-** | **-** | **-** |
| 78 | male | 85 | sputum | bilateral pneumonia | *A. baumannii* | R | R | **+** | **-** | **-** | **-** | **+** | **-** | **-** | **-** |
| 79 | male | 81 | sputum | brain infarction | *A. baumannii* | R | R | **+** | **-** | **-** | **-** | **+** | **-** | **-** | **-** |
| 80 | male | 87 | sputum | pneumonia | *P. aeruginosa* | R | R | **+** | **-** | **-** | **-** | **-** | **-** | **-** | **-** |
| 81 | male | 92 | sputum | chronic heart failure | *P. aeruginosa* | R | R | **+** | **-** | **-** | **-** | **-** | **-** | **-** | **-** |
| 82 | male | 90 | sputum | atrophic gastritis | *P. aeruginosa* | R | R | **+** | **-** | **-** | **-** | **-** | **-** | **-** | **-** |
| 83 | male | 88 | sputum | coronary artery disease | *P. aeruginosa* | R | R | **-** | **+** | **-** | **-** | **-** | **-** | **-** | **-** |
| 84 | male | 87 | sputum | diabetes mellitus type 2 | *P. aeruginosa* | R | R | **+** | **-** | **-** | **-** | **-** | **-** | **-** | **-** |
| 85 | male | 86 | sputum | cerebral infarction sequelae | *P. aeruginosa* | R | R | **-** | **-** | **-** | **-** | **-** | **-** | **-** | **-** |
| 86 | male | 90 | sputum | COPD | *P. aeruginosa* | R | R | **-** | **-** | **-** | **-** | **-** | **-** | **-** | **-** |
| 87 | male | 94 | sputum | pancreatic carcer | *P. aeruginosa* | R | R | **-** | **+** | **-** | **-** | **-** | **-** | **-** | **-** |
| 88 | male | 88 | sputum | dementia | *P. aeruginosa* | R | R | **+** | **-** | **-** | **-** | **-** | **-** | **-** | **-** |
| 89 | male | 94 | sputum | brain infarction | *P. aeruginosa* | R | R | **+** | **-** | **-** | **-** | **-** | **-** | **-** | **-** |
| 90 | male | 89 | sputum | cerebral infarction | *P. aeruginosa* | R | R | **+** | **-** | **-** | **-** | **-** | **-** | **-** | **-** |
| 91 | male | 90 | sputum | radiation enteritis | *P. aeruginosa* | R | R | **+** | **-** | **-** | **-** | **-** | **-** | **-** | **-** |
| 92 | male | 95 | sputum | cerebral hemorrhage sequela | *P. aeruginosa* | R | R | **+** | **-** | **-** | **-** | **-** | **-** | **-** | **-** |
| 93 | male | 90 | sputum | bilateral pneumonia | *P. aeruginosa* | R | R | **+** | **-** | **-** | **-** | **+** | **-** | **-** | **-** |
| 94 | male | 98 | sputum | pneumonia | *P. aeruginosa* | R | R | **+** | **-** | **-** | **-** | **+** | **-** | **-** | **-** |
| 95 | female | 96 | sputum | pneumonia | *P. aeruginosa* | R | S | **+** | **-** | **-** | **-** | **+** | **-** | **-** | **-** |
| 96 | male | 94 | sputum | bilateral pneumonia | *P. aeruginosa* | S | S | **-** | **-** | **-** | **-** | **-** | **-** | **-** | **-** |
| 97 | male | 90 | sputum | COPD | *E. coli* | S | S | **-** | **-** | **-** | **-** | **-** | **-** | **-** | **-** |
| 98 | male | 86 | sputum | cholecystitis | *K. pneumoniae* | R | S | **+** | **-** | **-** | **-** | **+** | **-** | **-** | **-** |
| 99 | male | 95 | sputum | pneumonia | *E. coli* | R | S | **+** | **-** | **-** | **-** | **-** | **-** | **-** | **-** |
| 100 | male | 95 | sputum | bilateral pneumonia | *K. pneumoniae* | R | R | **+** | **-** | **-** | **-** | **-** | **-** | **-** | **-** |
| 101 | male | 87 | sputum | bilateral pneumonia | *K. pneumoniae* | R | R | **+** | **-** | **-** | **-** | **-** | **-** | **-** | **-** |
| 102 | male | 86 | sputum | pneumonia | *K. pneumoniae* | S | R | **-** | **-** | **-** | **-** | **+** | **-** | **-** | **-** |
| 103 | male | 88 | sputum | bilateral pneumonia | *P. aeruginosa* | R | R | **+** | **-** | **-** | **-** | **+** | **-** | **-** | **-** |
| 104 | male | 95 | sputum | bilateral pneumonia | *P. aeruginosa* | S | S | **-** | **-** | **-** | **-** | **-** | **-** | **-** | **-** |
| 105 | male | 85 | sputum | bilateral pneumonia | *P. aeruginosa* | R | R | **+** | **-** | **-** | **-** | **+** | **-** | **-** | **-** |
| 106 | male | 94 | sputum | pneumonia | *P. aeruginosa* | R | R | **+** | **-** | **-** | **-** | **-** | **-** | **-** | **-** |
| 107 | male | 96 | sputum | pneumonia | *P. aeruginosa* | R | R | **+** | **-** | **-** | **-** | **-** | **-** | **-** | **-** |
| 108 | male | 82 | sputum | pneumonia | *P. aeruginosa* | S | S | **-** | **-** | **-** | **-** | **-** | **-** | **-** | **-** |
| 109 | male | 101 | sputum | seborrheic keratosis | *P. aeruginosa* | S | S | **-** | **-** | **-** | **-** | **-** | **-** | **-** | **-** |
| 110 | male | 89 | sputum | fracture | *K. pneumoniae* | R | R | **-** | **-** | **-** | **-** | **-** | **-** | **-** | **-** |
| 111 | male | 95 | sputum | bilateral pneumonia | *E. coli* | S | S | **-** | **-** | **-** | **-** | **-** | **-** | **-** | **-** |
| 112 | male | 87 | sputum | bilateral pneumonia | *E. coli* | S | R | **+** | **-** | **-** | **-** | **+** | **-** | **-** | **-** |
| 113 | male | 90 | sputum | pneumonia | *K. pneumoniae* | R | R | **-** | **-** | **-** | **-** | **-** | **-** | **-** | **-** |
| 114 | male | 89 | sputum | cerebral infarction | *E. coli* | S | S | **-** | **-** | **-** | **-** | **-** | **-** | **-** | **-** |
| 115 | male | 93 | sputum | pneumonia | *E. coli* | S | S | **-** | **-** | **-** | **-** | **-** | **-** | **-** | **-** |
| 116 | male | 93 | sputum | pneumonia | *Enterobacter aerogenes* | S | S | **-** | **-** | **-** | **-** | **-** | **-** | **-** | **-** |
| 117 | male | 89 | sputum | pneumonia | *A. baumannii* | R | R | **-** | **-** | **+** | **-** | **+** | **-** | **-** | **-** |
| 118 | male | 87 | sputum | intrahepatic cholangiocarcinoma | *P. aeruginosa* | I | S | **+** | **-** | **-** | **-** | **-** | **-** | **-** | **-** |
| 119 | male | 90 | sputum | bilateral pneumonia | *P. aeruginosa* | R | R | **-** | **-** | **-** | **-** | **-** | **-** | **-** | **-** |
| 120 | male | 86 | sputum | lower-lobe pneumonia | *E. coli* | S | S | **-** | **-** | **-** | **-** | **-** | **-** | **-** | **-** |
| 121 | female | 98 | sputum | chronic myelomonocytic leukaemia | *K. pneumoniae* | R | R | **+** | **-** | **-** | **-** | **-** | **-** | **-** | **-** |
| 122 | male | 86 | sputum | pneumonia | *K. pneumoniae* | R | R | **+** | **-** | **-** | **-** | **-** | **-** | **-** | **-** |
| 123 | male | 82 | sputum | advanced lung cancer | *K. pneumoniae* | R | R | **+** | **-** | **-** | **-** | **-** | **-** | **-** | **-** |
| 124 | male | 97 | sputum | pneumonia | *K. pneumoniae* | R | R | **+** | **-** | **-** | **-** | **+** | **-** | **-** | **-** |
| 125 | male | 85 | sputum | coronary artery disease | *Klebsiella oxytoca* | S | S | **-** | **-** | **-** | **-** | **-** | **-** | **-** | **-** |
| 126 | male | 86 | sputum | cerebral infarction sequelae | *P. aeruginosa* | S | S | **-** | **-** | **-** | **-** | **-** | **-** | **-** | **-** |
| 127 | male | 93 | sputum | bilateral pneumonia | *P. aeruginosa* | S | R | **+** | **-** | **-** | **-** | **+** | **-** | **-** | **-** |
| 128 | male | 91 | sputum | bilateral pneumonia | *P. aeruginosa* | R | R | **+** | **-** | **-** | **-** | **-** | **-** | **-** | **-** |
| 129 | male | 88 | sputum | cerebral infarction sequelae | *P. aeruginosa* | R | R | **+** | **-** | **-** | **-** | **-** | **-** | **-** | **-** |
| 130 | male | 79 | sputum | vascular dementia | *P. aeruginosa* | R | R | **+** | **-** | **-** | **-** | **-** | **-** | **-** | **-** |
| 131 | male | 79 | sputum | Parkinson's disease | *P. aeruginosa* | R | R | **+** | **-** | **-** | **-** | **-** | **-** | **-** | **-** |
| 132 | male | 83 | sputum | hypertension (grade III) | *P. aeruginosa* | R | R | **+** | **-** | **-** | **-** | **-** | **-** | **-** | **-** |
| 133 | male | 90 | sputum | chronic bronchitis | *P. aeruginosa* | R | R | **+** | **-** | **-** | **-** | **+** | **-** | **-** | **-** |
| 134 | female | 90 | sputum | COPD | *A. baumannii* | R | R | **+** | **-** | **-** | **-** | **+** | **-** | **-** | **-** |
| 135 | male | 86 | sputum | bilateral pneumonia | *K. pneumoniae* | R | R | **+** | **-** | **-** | **-** | **-** | **-** | **-** | **-** |
| 136 | male | 88 | sputum | COPD | *P. aeruginosa* | R | R | **+** | **-** | **-** | **-** | **+** | **-** | **-** | **-** |
| 137 | female | 91 | sputum | pneumonia, gastrointestinal dysfunction | *P. aeruginosa* | R | R | **+** | **-** | **-** | **-** | **-** | **-** | **-** | **-** |
| 138 | male | 93 | sputum | pneumonia | *P. aeruginosa* | R | R | **+** | **-** | **-** | **-** | **+** | **-** | **-** | **-** |
| 139 | male | 90 | sputum | radiation enteritis | *P. aeruginosa* | R | R | **-** | **-** | **-** | **-** | **-** | **-** | **-** | **-** |
| 140 | male | 94 | sputum | pulmonary infection | *P. aeruginosa* | R | R | **-** | **-** | **-** | **-** | **-** | **-** | **-** | **-** |
| 141 | male | 96 | sputum | Pneumonia, COPD | *P. aeruginosa* | R | R | **+** | **-** | **-** | **-** | **+** | **-** | **-** | **-** |
| 142 | male | 92 | sputum | bilateral pneumonia | *P. aeruginosa* | R | R | **+** | **+** | **-** | **-** | **-** | **-** | **-** | **-** |
| 143 | male | 87 | sputum | pneumonia | *P. aeruginosa* | R | R | **+** | **-** | **-** | **-** | **+** | **-** | **-** | **-** |
| 144 | male | 90 | sputum | pneumonia,COPD | *P. aeruginosa* | R | R | **+** | **-** | **-** | **-** | **-** | **-** | **-** | **-** |
| 145 | male | 86 | sputum | advanced lung cancer | *P. aeruginosa* | R | R | **+** | **+** | **-** | **-** | **+** | **-** | **-** | **-** |
| 146 | male | 92 | sputum | cerebral infarctionsequelae | *P. aeruginosa* | I | I | **+** | **+** | **-** | **-** | **+** | **-** | **-** | **-** |
| 147 | male | 86 | sputum | brain infarctionsequelae | *P. aeruginosa* | S | S | **-** | **-** | **-** | **-** | **-** | **-** | **-** | **-** |
| 148 | male | 90 | sputum | COPD | *P. aeruginosa* | R | R | **+** | **-** | **-** | **-** | **-** | **-** | **-** | **-** |
| 149 | male | 90 | sputum | pneumonia | *A. baumannii* | R | R | **+** | **-** | **+** | **-** | **+** | **-** | **-** | **-** |
| 150 | male | 90 | sputum | cerebral hemorrhage | *A. baumannii* | R | R | **+** | **-** | **+** | **-** | **+** | **-** | **-** | **-** |
| 151 | male | 87 | sputum | bilateral pneumonia | *A. baumannii* | R | R | **+** | **-** | **+** | **-** | **+** | **-** | **-** | **-** |
| 152 | male | 87 | sputum | pneumonia | *P. aeruginosa* | R | R | **+** | **-** | **-** | **-** | **+** | **-** | **-** | **-** |
| 153 | male | 88 | sputum | chronic heart failure | *P. aeruginosa* | R | R | **-** | **-** | **-** | **-** | **-** | **-** | **-** | **-** |
| 154 | male | 89 | sputum | pneumonia | *P. aeruginosa* | R | R | **+** | **-** | **-** | **-** | **-** | **-** | **-** | **-** |
| 155 | male | 90 | sputum | COPD | *P. aeruginosa* | R | R | **+** | **-** | **-** | **-** | **+** | **-** | **-** | **-** |
| 156 | male | 89 | sputum | cerebral infarction | *E. coli* | S | R | **-** | **-** | **+** | **-** | **+** | **-** | **-** | **-** |
| 157 | male | 94 | sputum | bilateral pneumonia | *Achromobacter xylosoxidans* | R | R | **+** | **-** | **-** | **-** | **+** | **-** | **-** | **-** |
| 158 | male | 89 | sputum | pneumonia | *A. baumannii* | R | R | **+** | **+** | **-** | **-** | **+** | **-** | **-** | **-** |
| 159 | male | 86 | sputum | bilateral pneumonia | *P. aeruginosa* | R | R | **-** | **+** | **-** | **-** | **+** | **-** | **-** | **-** |
| 160 | male | 88 | sputum | bilateral pneumonia | *P. aeruginosa* | R | R | **+** | **-** | **-** | **-** | **-** | **-** | **-** | **-** |
| 161 | male | 89 | sputum | arrhythmia | *P. aeruginosa* | S | S | **-** | **-** | **-** | **-** | **-** | **-** | **-** | **-** |
| 162 | male | 95 | sputum | chronic bronchitis | *P. aeruginosa* | R | R | **+** | **-** | **-** | **-** | **+** | **-** | **-** | **-** |
| 163 | male | 89 | sputum | cerebral infarction sequelae | *P. aeruginosa* | R | R | **+** | **-** | **+** | **-** | **+** | **-** | **-** | **-** |
| 164 | male | 98 | sputum | pneumonia | *A. baumannii* | R | R | **-** | **-** | **+** | **-** | **+** | **-** | **-** | **-** |
| 165 | male | 95 | sputum | pneumonia | *A. baumannii* | R | R | **+** | **+** | **+** | **-** | **+** | **-** | **-** | **-** |
| 166 | male | 75 | sputum | pneumonia | *P. aeruginosa* | R | R | **+** | **-** | **-** | **-** | **+** | **-** | **-** | **-** |
| 167 | male | 92 | sputum | pneumonia | *P. aeruginosa* | I | S | **-** | **-** | **-** | **-** | **-** | **-** | **-** | **-** |
| 168 | male | 95 | sputum | coronary artery disease | *P. aeruginosa* | R | R | **+** | **-** | **-** | **-** | **-** | **-** | **-** | **-** |
| 169 | male | 91 | sputum | advanced lung cancer | *P. aeruginosa* | R | R | **-** | **-** | **-** | **-** | **+** | **-** | **-** | **-** |
| 170 | male | 85 | sputum | bilateral pneumonia | *P. aeruginosa* | R | R | **+** | **-** | **-** | **-** | **-** | **-** | **-** | **-** |
| 171 | male | 95 | sputum | cerebral hemorrhage | *P. aeruginosa* | R | R | **+** | **-** | **-** | **-** | **+** | **-** | **-** | **-** |
| 172 | female | 72 | sputum | gastritis | *K. pneumoniae* | S | S | **-** | **-** | **-** | **-** | **-** | **-** | **-** | **-** |
| 173 | male | 89 | sputum | fracture | *P. aeruginosa* | R | R | **+** | **-** | **-** | **-** | **+** | **-** | **-** | **-** |
| 174 | male | 88 | sputum | COPD | *P. aeruginosa* | R | R | **+** | **-** | **-** | **-** | **+** | **-** | **-** | **-** |
| 175 | male | 86 | sputum | pneumonia | *A. baumannii* | R | R | **+** | **-** | **-** | **-** | **+** | **-** | **-** | **-** |
| 176 | male | 92 | sputum | cerebral infarction | *A. baumannii* | R | R | **-** | **-** | **+** | **-** | **+** | **-** | **-** | **-** |
| 177 | male | 93 | sputum | pneumonia | *P. aeruginosa* | R | R | **+** | **-** | **-** | **-** | **+** | **-** | **-** | **-** |
| 178 | male | 87 | urine | urinary infection | *P. aeruginosa* | R | R | **+** | **-** | **-** | **-** | **+** | **-** | **-** | **-** |
| 179 | male | 87 | sputum | pneumonia | *E. coli* | S | S | **-** | **-** | **-** | **-** | **-** | **-** | **-** | **-** |
| 180 | male | 90 | sputum | pneumonia | *A. baumannii* | R | R | **-** | **-** | **+** | **-** | **+** | **-** | **-** | **-** |
| 181 | male | 102 | sputum | pneumonia | *P. aeruginosa* | S | R | **+** | **-** | **+** | **-** | **+** | **-** | **-** | **-** |
| 182 | male | 96 | sputum | pneumonia，COPD | *P. aeruginosa* | R | R | **-** | **+** | **-** | **-** | **-** | **-** | **-** | **-** |
| 183 | male | 85 | sputum | brain infarction | *P. aeruginosa* | S | S | **-** | **-** | **-** | **-** | **-** | **-** | **-** | **-** |
| 184 | male | 94 | sputum | Alzheimer disease | *P. aeruginosa* | R | R | **+** | **-** | **-** | **-** | **-** | **-** | **-** | **-** |
| 185 | male | 99 | sputum | intestinal function disorder | *K. pneumoniae* | S | S | **-** | **-** | **-** | **-** | **-** | **-** | **-** | **-** |
| 186 | male | 89 | sputum | fracture | *Serratia marcescens* | S | S | **-** | **-** | **-** | **-** | **-** | **-** | **-** | **-** |
| 187 | male | 89 | sputum | fracture | *K. pneumoniae* | R | R | **+** | **-** | **-** | **-** | **-** | **-** | **-** | **-** |
| 188 | male | 96 | sputum | pneumonia | *K. pneumoniae* | R | R | **+** | **-** | **-** | **-** | **-** | **-** | **-** | **-** |
| 189 | male | 87 | sputum | chronic myelomonocytic leukemia | *P. aeruginosa* | I | S | **+** | **-** | **-** | **-** | **-** | **-** | **-** | **-** |
| 190 | male | 93 | sputum | hypertension | *P. aeruginosa* | S | S | **-** | **-** | **-** | **-** | **-** | **-** | **-** | **-** |
| 191 | male | 86 | sputum | cerebral infarction sequelae | *P. aeruginosa* | R | R | **-** | **-** | **-** | **-** | **+** | **-** | **-** | **-** |
| 192 | male | 95 | sputum | bilateral pneumonia | *A. baumannii* | R | R | **-** | **-** | **+** | **-** | **+** | **-** | **-** | **-** |
| 193 | male | 89 | sputum | cerebral infarction sequelae | *A. baumannii* | R | R | **-** | **-** | **+** | **-** | **+** | **-** | **-** | **-** |
| 194 | male | 90 | sputum | pneumonia, arrhythmia | *P. aeruginosa* | S | S | **-** | **-** | **-** | **-** | **-** | **-** | **-** | **-** |
| 195 | male | 91 | sputum | pneumonia | *P. aeruginosa* | S | S | **-** | **-** | **-** | **-** | **-** | **-** | **-** | **-** |
| 196 | male | 93 | sputum | hypertension | *P. aeruginosa* | S | S | **-** | **-** | **-** | **-** | **-** | **-** | **-** | **-** |
| 197 | male | 92 | sputum | bilateral pneumonia | *P. aeruginosa* | R | R | **-** | **-** | **-** | **-** | **-** | **-** | **-** | **+** |
| 198 | male | 96 | sputum | pneumonia | *Serratia marcescens* | S | S | **-** | **-** | **-** | **-** | **-** | **-** | **-** | **-** |
| 199 | male | 100 | sputum | bilateral pneumonia | *E. coli* | S | S | **-** | **-** | **-** | **-** | **-** | **-** | **-** | **-** |
| 200 | female | 98 | sputum | chronic myelomonocytic leukaemia | *K. pneumoniae* | R | R | **+** | **-** | **-** | **-** | **-** | **-** | **-** | **-** |
| 201 | male | 86 | sputum | pneumonia | *K. pneumoniae* | R | R | **+** | **-** | **-** | **-** | **-** | **-** | **-** | **-** |
| 202 | male | 86 | sputum | bilateral pneumonia | *E. coli* | S | S | **-** | **-** | **-** | **-** | **-** | **-** | **-** | **-** |
| 203 | male | 88 | sputum | bilateral pneumonia | *K. pneumoniae* | R | R | **+** | **-** | **-** | **-** | **-** | **-** | **-** | **-** |
| 204 | male | 97 | sputum | pneumonia | *K. pneumoniae* | R | R | **+** | **-** | **-** | **-** | **-** | **-** | **-** | **-** |
| 205 | male | 90 | sputum | Parkinson’s disease | *P. aeruginosa* | R | R | **+** | **-** | **-** | **-** | **+** | **-** | **-** | **-** |
| 206 | male | 89 | sputum | bilateral pneumonia | *P. aeruginosa* | R | R | **+** | **-** | **-** | **-** | **+** | **-** | **-** | **-** |
| 207 | male | 95 | sputum | pulmonary infection | *P. aeruginosa* | R | R | **-** | **-** | **-** | **+** | **+** | **-** | **-** | **-** |
| 208 | male | 91 | sputum | bilateral pneumonia | *P. aeruginosa* | R | R | **+** | **-** | **-** | **-** | **-** | **-** | **-** | **-** |
| 209 | male | 96 | sputum | cerebral infarction sequelae | *P. aeruginosa* | R | R | **+** | **-** | **-** | **-** | **+** | **-** | **-** | **-** |
| 210 | male | 90 | sputum | radiation enteritis | *P. aeruginosa* | R | R | **+** | **-** | **-** | **-** | **-** | **-** | **-** | **-** |
| 211 | male | 90 | sputum | COPD | *P. aeruginosa* | R | R | **+** | **-** | **-** | **-** | **-** | **-** | **-** | **-** |
| 212 | male | 90 | urine | COPD | *E. coli* | S | S | **-** | **-** | **-** | **-** | **-** | **-** | **-** | **-** |
| 213 | male | 88 | sputum | pneumonia | *E. coli* | S | S | **-** | **-** | **-** | **-** | **-** | **-** | **-** | **-** |
| 214 | male | 98 | sputum | bilateral pneumonia | *E. coli* | S | S | **-** | **-** | **-** | **-** | **-** | **-** | **-** | **-** |
| 215 | male | 86 | sputum | cerebral infarction | *Serratia marcescens* | S | S | **-** | **-** | **-** | **-** | **-** | **-** | **-** | **-** |
| 216 | male | 90 | sputum | COPD | *P. aeruginosa* | R | R | **+** | **-** | **-** | **-** | **-** | **-** | **-** | **-** |
| 217 | male | 93 | sputum | bilateral pneumonia | *P. aeruginosa* | R | R | **-** | **-** | **-** | **-** | **-** | **-** | **-** | **-** |
| 218 | male | 87 | sputum | pneumonia | *P. aeruginosa* | R | R | **+** | **-** | **-** | **-** | **-** | **-** | **-** | **-** |
| 219 | male | 101 | sputum | pneumonia | *P. aeruginosa* | R | R | **-** | **-** | **-** | **-** | **+** | **-** | **-** | **-** |
| 220 | male | 98 | sputum | pulmonary infection | *P. aeruginosa* | R | R | **+** | **+** | **-** | **-** | **-** | **-** | **-** | **-** |
| 221 | male | 94 | sputum | pneumonia | *P. aeruginosa* | S | R | **+** | **-** | **-** | **-** | **-** | **-** | **-** | **-** |
| 222 | male | 86 | sputum | pneumonia | *P. aeruginosa* | S | R | **+** | **-** | **-** | **-** | **+** | **-** | **-** | **-** |
| 223 | male | 94 | sputum | ventilator associated pneumonia | *P. aeruginosa* | R | R | **+** | **-** | **-** | **-** | **+** | **-** | **-** | **-** |
| 224 | male | 92 | sputum | cerebral infarction | *P. aeruginosa* | R | R | **+** | **-** | **-** | **-** | **-** | **-** | **-** | **-** |
| 225 | male | 97 | sputum | bilateral pneumonia | *P. aeruginosa* | R | R | **+** | **-** | **-** | **-** | **+** | **-** | **-** | **-** |
| 226 | male | 96 | sputum | cerebral infarction sequelae | *P. aeruginosa* | R | R | **+** | **-** | **-** | **-** | **-** | **-** | **-** | **-** |
| 227 | male | 95 | sputum | bilateral pneumonia | *P. aeruginosa* | R | R | **+** | **-** | **-** | **-** | **-** | **-** | **-** | **-** |
| 228 | male | 90 | sputum | bilateral pneumonia | *K. pneumoniae* | R | R | **+** | **-** | **-** | **-** | **-** | **-** | **-** | **-** |
| 229 | male | 90 | sputum | cerebral infarction sequelae | *E. coli* | S | S | **-** | **-** | **-** | **-** | **-** | **-** | **-** | **-** |
| 230 | male | 85 | sputum | subdural haematoma | *K. pneumoniae* | S | S | **-** | **-** | **-** | **-** | **-** | **-** | **-** | **-** |
| 231 | male | 95 | sputum | pulmonary infection | *K. pneumoniae* | R | R | **+** | **-** | **-** | **-** | **-** | **-** | **-** | **-** |
| 232 | male | 92 | sputum | bilateral pneumonia | *K. pneumoniae* | R | R | **+** | **-** | **-** | **-** | **-** | **-** | **-** | **-** |
| 233 | male | 87 | sputum | intrahepatic cholangiocarcinoma | *K. pneumoniae* | R | R | **+** | **-** | **-** | **-** | **-** | **-** | **-** | **-** |
| 234 | male | 90 | sputum | radiation enteritis | *P. aeruginosa* | R | R | **+** | **-** | **-** | **-** | **-** | **-** | **-** | **-** |
| 235 | male | 86 | sputum | pneumonia | *P. aeruginosa* | R | R | **+** | **-** | **-** | **-** | **-** | **-** | **-** | **-** |
| 236 | male | 90 | sputum | Parkinson’s disease | *P. aeruginosa* | R | R | **+** | **-** | **-** | **-** | **+** | **-** | **-** | **-** |
| 237 | male | 87 | sputum | angina pectoris | *P. aeruginosa* | R | R | **+** | **-** | **-** | **-** | **-** | **-** | **-** | **-** |
| 238 | male | 93 | sputum | bilateral pneumonia | *P. aeruginosa* | R | R | **+** | **-** | **-** | **-** | **-** | **-** | **-** | **-** |
| 239 | male | 89 | sputum | pneumonia | *A. baumannii* | R | R | **-** | **-** | **+** | **-** | **+** | **-** | **-** | **-** |
| 240 | male | 86 | sputum | pneumonia | *K. pneumoniae* | R | R | **+** | **-** | **-** | **-** | **-** | **-** | **-** | **-** |
| 241 | male | 93 | sputum | hypertension | *K. pneumoniae* | S | S | **-** | **-** | **-** | **-** | **-** | **-** | **-** | **-** |
| 242 | male | 86 | sputum | bilateral pneumonia | *E. coli* | S | S | **-** | **-** | **-** | **-** | **-** | **-** | **-** | **-** |
| 243 | male | 94 | sputum | bilateral pneumonia | *E. coli* | S | S | **-** | **-** | **-** | **-** | **-** | **-** | **-** | **-** |
| 244 | male | 86 | urine | pneumonia | *Flavobacterium meningosepticum* | R | R | **+** | **-** | **-** | **-** | **-** | **-** | **-** | **-** |
| 245 | male | 87 | sputum | bilateral pneumonia | *A. baumannii* | R | R | **-** | **-** | **-** | **-** | **+** | **+** | **-** | **-** |
| 246 | male | 97 | sputum | pneumonia | *K. pneumoniae* | R | R | **-** | **-** | **-** | **-** | **-** | **-** | **-** | **-** |
| 247 | male | 89 | sputum | cerebral infarction | *E. coli* | S | S | **-** | **-** | **-** | **-** | **-** | **-** | **-** | **-** |
| 248 | male | 59 | urine | urinary infection | *Enterobacter cloacae* | S | S | **-** | **-** | **-** | **-** | **-** | **-** | **-** | **-** |
| 249 | male | 95 | sputum | bilateral pneumonia | *K. pneumoniae* | S | S | **-** | **-** | **-** | **-** | **-** | **-** | **-** | **-** |
| 250 | female | 98 | sputum | chronic myelomonocytic leukaemia | *K. pneumoniae* | R | R | **+** | **-** | **-** | **-** | **-** | **-** | **-** | **-** |
| 251 | male | 95 | sputum | craniocerebral trauma | *K. pneumoniae* | R | R | **+** | **-** | **-** | **-** | **-** | **-** | **-** | **-** |
| 252 | male | 93 | sputum | pneumonia | *P. aeruginosa* | R | R | **-** | **-** | **-** | **-** | **-** | **-** | **-** | **-** |
| 253 | male | 93 | sputum | pneumonia | *P. aeruginosa* | R | R | **+** | **-** | **-** | **-** | **-** | **-** | **-** | **-** |
| 254 | male | 91 | sputum | pneumonia | *P. aeruginosa* | S | S | **-** | **-** | **-** | **-** | **-** | **-** | **-** | **-** |
| 255 | male | 90 | sputum | bilateral pneumonia | *P. aeruginosa* | R | R | **+** | **-** | **-** | **-** | **-** | **-** | **-** | **-** |
| 256 | male | 86 | sputum | pneumonia | *P. aeruginosa* | S | S | **-** | **-** | **-** | **-** | **-** | **-** | **-** | **-** |
| 257 | male | 87 | sputum | bilateral pneumonia | *P. aeruginosa* | S | S | **-** | **-** | **-** | **-** | **-** | **-** | **-** | **-** |
| 258 | male | 93 | sputum | bilateral pneumonia | *P. aeruginosa* | R | I | **+** | **-** | **-** | **-** | **-** | **-** | **-** | **-** |
| 259 | male | 88 | sputum | COPD | *P. aeruginosa* | R | R | **+** | **-** | **-** | **-** | **-** | **-** | **-** | **-** |
| 260 | male | 90 | sputum | pneumonia | *P. aeruginosa* | R | R | **+** | **-** | **-** | **-** | **-** | **-** | **-** | **-** |
| 261 | female | 91 | sputum | pneumonia，gastrointestinal dysfunction | *P. aeruginosa* | R | R | **-** | **-** | **-** | **-** | **-** | **-** | **-** | **-** |
| 262 | female | 95 | sputum | cerebral hemorrhage | *P. aeruginosa* | R | R | **+** | **+** | **-** | **-** | **-** | **-** | **-** | **-** |
| 263 | male | 89 | sputum | cerebral infarction sequelae | *A. baumannii* | R | R | **-** | **-** | **+** | **-** | **+** | **-** | **-** | **-** |
| 264 | male | 92 | sputum | bilateral pneumonia | *E. coli* | S | S | **-** | **-** | **-** | **-** | **-** | **-** | **-** | **-** |
| 265 | male | 100 | urine | bilateral pneumonia | *K. pneumoniae* | S | S | **-** | **-** | **-** | **-** | **-** | **-** | **-** | **-** |
| 266 | male | 94 | sputum | COPD | *P. aeruginosa* | R | R | **+** | **-** | **-** | **-** | **-** | **-** | **-** | **-** |
| 267 | male | 94 | sputum | ventilator associated pneumonia | *P. aeruginosa* | R | R | **+** | **-** | **-** | **-** | **-** | **-** | **-** | **-** |
| 268 | male | 91 | sputum | COPD, pneumonia | *P. aeruginosa* | R | R | **+** | **-** | **-** | **-** | **-** | **-** | **-** | **-** |
| 269 | male | 88 | sputum | pneumonia | *P. aeruginosa* | S | S | **-** | **-** | **-** | **-** | **-** | **-** | **-** | **-** |
| 270 | male | 87 | sputum | bilateral pneumonia | *P. aeruginosa* | R | R | **+** | **-** | **-** | **-** | **-** | **-** | **-** | **-** |
| 271 | male | 86 | sputum | bilateral pneumonia | *P. aeruginosa* | R | R | **+** | **-** | **-** | **-** | **-** | **-** | **-** | **-** |
| 272 | male | 89 | sputum | pneumonia | *P. aeruginosa* | R | R | **+** | **-** | **-** | **-** | **+** | **-** | **-** | **-** |
| 273 | male | 91 | sputum | bilateral pneumonia | *P. aeruginosa* | R | R | **+** | **-** | **-** | **-** | **+** | **-** | **-** | **-** |
| 274 | male | 90 | sputum | bilateral pneumonia | *P. aeruginosa* | R | R | **+** | **-** | **-** | **-** | **-** | **-** | **-** | **-** |
| 275 | male | 90 | sputum | pneumonia | *P. aeruginosa* | R | R | **+** | **-** | **-** | **-** | **+** | **-** | **-** | **-** |
| 276 | male | 85 | sputum | bilateral pneumonia | *P. aeruginosa* | R | R | **-** | **-** | **-** | **-** | **+** | **-** | **-** | **-** |
| 277 | male | 89 | sputum | chronic bronchitis | *P. aeruginosa* | R | R | **-** | **-** | **-** | **-** | **+** | **-** | **-** | **-** |
| 278 | female | 90 | sputum | pneumonia | *P. aeruginosa* | R | R | **-** | **-** | **-** | **-** | **+** | **-** | **-** | **-** |
| 279 | male | 86 | sputum | bilateral pneumonia | *Serratia marcescens* | S | S | **-** | **-** | **-** | **-** | **-** | **-** | **-** | **-** |
| 280 | male | 98 | sputum | pneumonia | *P. aeruginosa* | R | R | **+** | **-** | **-** | **-** | **-** | **-** | **-** | **-** |
| 281 | female | 93 | sputum | bilateral pneumonia | *A. baumannii* | R | R | **-** | **-** | **+** | **-** | **+** | **-** | **-** | **-** |
| 282 | male | 97 | sputum | bilateral pneumonia | *P. aeruginosa* | R | R | **+** | **-** | **-** | **-** | **+** | **-** | **-** | **-** |
| 283 | male | 93 | urine | pneumonia | *P. aeruginosa* | R | R | **+** | **-** | **-** | **-** | **-** | **-** | **-** | **-** |
| 284 | male | 91 | sputum | bilateral pneumonia | *P. aeruginosa* | R | R | **+** | **-** | **-** | **-** | **-** | **-** | **-** | **-** |
| 285 | female | 90 | sputum | pneumonia | *P. aeruginosa* | R | R | **+** | **-** | **-** | **-** | **-** | **-** | **-** | **-** |
| 286 | male | 93 | sputum | bilateral pneumonia | *P. aeruginosa* | R | I | **+** | **-** | **-** | **-** | **+** | **-** | **-** | **-** |
| 287 | male | 86 | sputum | pneumonia | *P. aeruginosa* | S | S | **-** | **-** | **-** | **-** | **-** | **-** | **-** | **-** |
| 288 | male | 95 | sputum | bilateral pneumonia | *P. aeruginosa* | S | S | **-** | **-** | **-** | **-** | **-** | **-** | **-** | **-** |
| 289 | male | 90 | sputum | chronic bronchitis | *P. aeruginosa* | R | R | **+** | **-** | **-** | **-** | **-** | **-** | **-** | **-** |
| 290 | male | 88 | sputum | bilateral pneumonia | *P. aeruginosa* | R | R | **+** | **-** | **-** | **-** | **-** | **-** | **-** | **-** |
| 291 | male | 93 | sputum | pulmonary infection | *P. aeruginosa* | R | R | **+** | **-** | **-** | **-** | **-** | **-** | **-** | **-** |
| 292 | male | 102 | sputum | pneumonia | *P. aeruginosa* | R | I | **+** | **-** | **-** | **-** | **-** | **-** | **-** | **-** |
| 293 | male | 88 | sputum | chronic bronchitis | *P. aeruginosa* | R | R | **+** | **-** | **-** | **-** | **+** | **-** | **-** | **-** |
| 294 | male | 93 | sputum | pneumonia | *P. aeruginosa* | R | R | **+** | **-** | **-** | **-** | **+** | **-** | **-** | **-** |
| 295 | male | 93 | sputum | pneumonia | *P. aeruginosa* | R | R | **+** | **-** | **-** | **-** | **+** | **-** | **-** | **-** |
| 296 | male | 91 | sputum | bilateral pneumonia | *P. aeruginosa* | S | S | **-** | **-** | **-** | **-** | **-** | **-** | **-** | **-** |
| 297 | male | 98 | sputum | pneumonia | *P. aeruginosa* | R | R | **+** | **-** | **-** | **-** | **-** | **-** | **-** | **-** |
| 298 | male | 87 | sputum | pulmonary infection | *P. aeruginosa* | S | S | **-** | **-** | **-** | **-** | **-** | **-** | **-** | **-** |
| 299 | male | 96 | sputum | Pneumonia, COPD | *P. aeruginosa* | R | R | **+** | **-** | **-** | **-** | **-** | **-** | **-** | **-** |
| 300 | male | 89 | sputum | chronic bronchitis | *P. aeruginosa* | R | R | **+** | **-** | **-** | **-** | **+** | **-** | **-** | **-** |
| 301 | male | 89 | sputum | pneumonia | *A. baumannii* | R | R | **+** | **-** | **+** | **-** | **+** | **-** | **-** | **-** |
| 302 | male | 92 | sputum | bilateral pneumonia | *A. baumannii* | S | S | **-** | **-** | **-** | **-** | **-** | **-** | **-** | **-** |
| 303 | male | 59 | sputum | bilateral pneumonia | *Flavobacterium meningosepticum* | R | R | **+** | **-** | **-** | **-** | **-** | **-** | **-** | **-** |
| 304 | male | 100 | sputum | bilateral pneumonia | *E. coli* | S | S | **-** | **-** | **-** | **-** | **-** | **-** | **-** | **-** |
| 305 | female | 62 | urine | urinary infection | *E. coli* | S | S | **-** | **-** | **-** | **-** | **-** | **-** | **-** | **-** |
| 306 | male | 97 | sputum | pneumonia | *K. pneumoniae* | R | R | **+** | **-** | **-** | **-** | **-** | **-** | **-** | **-** |
| 307 | male | 96 | sputum | pneumonia | *K. pneumoniae* | R | R | **+** | **-** | **-** | **-** | **-** | **-** | **-** | **-** |
| 308 | male | 85 | sputum | bilateral pneumonia | *K. pneumoniae* | R | R | **+** | **-** | **-** | **-** | **+** | **-** | **-** | **-** |
| 309 | male | 94 | sputum | pneumonia | *P. aeruginosa* | R | I | **+** | **-** | **-** | **-** | **-** | **-** | **-** | **-** |
| 310 | female | 91 | sputum | pneumonia，gastrointestinal dysfunction | *P. aeruginosa* | R | R | **+** | **-** | **-** | **-** | **+** | **-** | **-** | **-** |
| 311 | male | 87 | sputum | bilateral pneumonia | *P. aeruginosa* | S | S | **-** | **-** | **-** | **-** | **-** | **-** | **-** | **-** |
| 312 | male | 88 | sputum | chronic heart failure | *P. aeruginosa* | R | R | **+** | **-** | **-** | **-** | **+** | **-** | **-** | **-** |
| 313 | male | 88 | urine | pneumonia | *P. aeruginosa* | S | S | **-** | **-** | **-** | **-** | **-** | **-** | **-** | **-** |
| 314 | male | 87 | sputum | advanced lung cancer | *P. aeruginosa* | R | R | **+** | **-** | **-** | **-** | **-** | **-** | **-** | **-** |
| 315 | male | 90 | sputum | Parkinson’s disease | *P. aeruginosa* | R | R | **+** | **-** | **-** | **-** | **+** | **-** | **-** | **-** |
| 316 | male | 89 | sputum | bilateral pneumonia | *P. aeruginosa* | R | R | **+** | **-** | **-** | **-** | **-** | **-** | **-** | **-** |
| 317 | male | 90 | sputum | radiation enteritis | *P. aeruginosa* | R | R | **+** | **-** | **-** | **-** | **-** | **-** | **-** | **-** |
| 318 | male | 92 | sputum | cerebral infarction sequelae | *P. aeruginosa* | R | R | **+** | **-** | **-** | **-** | **-** | **-** | **-** | **-** |
| 319 | male | 91 | sputum | bilateral pneumonia | *P. aeruginosa* | R | R | **+** | **-** | **-** | **-** | **-** | **-** | **-** | **-** |
| 320 | male | 98 | sputum | pulmonary infection | *P. aeruginosa* | R | R | **+** | **-** | **-** | **-** | **-** | **-** | **-** | **-** |
| 321 | male | 95 | sputum | COPD | *Serratia marcescens* | S | S | **-** | **-** | **-** | **-** | **-** | **-** | **-** | **-** |
| 322 | male | 89 | sputum | pneumonia | *K. pneumoniae* | S | S | **-** | **-** | **-** | **-** | **-** | **-** | **-** | **-** |
| 323 | male | 94 | sputum | bilateral pneumonia | *K. pneumoniae* | R | R | **+** | **-** | **-** | **-** | **-** | **-** | **-** | **-** |
| 324 | male | 98 | sputum | COPD | *E. coli* | S | S | **-** | **-** | **-** | **-** | **-** | **-** | **-** | **-** |
| 325 | male | 88 | sputum | bilateral pneumonia | *P. aeruginosa* | S | S | **-** | **-** | **-** | **-** | **-** | **-** | **-** | **-** |
| 326 | male | 90 | sputum | pneumonia,COPD | *P. aeruginosa* | R | R | **+** | **-** | **-** | **-** | **-** | **-** | **-** | **-** |
| 327 | male | 94 | sputum | bilateral pneumonia | *P. aeruginosa* | R | R | **+** | **-** | **-** | **-** | **+** | **-** | **-** | **-** |
| 328 | male | 95 | sputum | bilateral pneumonia | *P. aeruginosa* | S | S | **-** | **-** | **-** | **-** | **-** | **-** | **-** | **-** |
| 329 | male | 88 | sputum | COPD | *P. aeruginosa* | R | R | **+** | **-** | **-** | **-** | **+** | **-** | **-** | **-** |
| 330 | male | 91 | sputum | bilateral pneumonia | *P. aeruginosa* | R | R | **+** | **-** | **-** | **-** | **-** | **-** | **-** | **-** |
| 331 | male | 92 | sputum | bilateral pneumonia | *P. aeruginosa* | R | R | **+** | **-** | **-** | **-** | **-** | **-** | **-** | **-** |
| 332 | male | 92 | sputum | bilateral pneumonia | *P. aeruginosa* | R | R | **+** | **-** | **-** | **-** | **-** | **-** | **-** | **-** |
| 333 | male | 88 | sputum | pneumonia | *P. aeruginosa* | R | R | **+** | **-** | **-** | **-** | **-** | **-** | **-** | **-** |
| 334 | male | 87 | sputum | diabetes mellitus type 2 | *P. aeruginosa* | R | R | **+** | **-** | **-** | **-** | **+** | **-** | **-** | **-** |
| 335 | male | 91 | sputum | COPD,pneumonia | *P. aeruginosa* | R | R | **+** | **-** | **-** | **-** | **+** | **-** | **-** | **-** |
| 336 | male | 90 | sputum | COPD | *P. aeruginosa* | R | R | **+** | **-** | **-** | **-** | **-** | **-** | **-** | **+** |
| 337 | male | 86 | sputum | cerebral infarction sequelae | *P. aeruginosa* | S | S | **-** | **-** | **-** | **-** | **-** | **-** | **-** | **-** |
| 338 | male | 91 | sputum | Parkinson’s disease | *P. aeruginosa* | S | S | **-** | **-** | **-** | **-** | **-** | **-** | **-** | **-** |
| 339 | male | 89 | sputum | cerebral infarction sequelae | *A. baumannii* | R | R | **-** | **-** | **+** | **-** | **+** | **-** | **-** | **-** |
| 340 | male | 94 | sputum | bilateral pneumonia | *P. aeruginosa* | S | S | **-** | **-** | **-** | **-** | **-** | **-** | **-** | **-** |
| 341 | male | 95 | sputum | Alzheimer disease | *P. aeruginosa* | R | R | **+** | **-** | **-** | **-** | **-** | **-** | **-** | **+** |
| 342 | male | 71 | sputum | pneumonia | *A. baumannii* | R | R | **-** | **-** | **-** | **+** | **-** | **-** | **-** | **-** |
| 343 | male | 87 | sputum | bilateral pneumonia | *Klebsiella oxytoca* | S | S | **-** | **-** | **-** | **-** | **-** | **-** | **-** | **-** |
| 344 | female | 93 | sputum | bilateral pneumonia | *E. coli* | S | S | **-** | **-** | **-** | **-** | **-** | **-** | **-** | **-** |
| 345 | male | 87 | sputum | intrahepatic cholangiocarcinoma | *E. coli* | S | S | **-** | **-** | **-** | **-** | **-** | **-** | **-** | **-** |
| 346 | male | 95 | sputum | pneumonia | *K. pneumoniae* | R | R | **+** | **-** | **-** | **-** | **-** | **-** | **-** | **-** |
| 347 | male | 95 | sputum | craniocerebral trauma | *K. pneumoniae* | R | R | **+** | **-** | **-** | **-** | **-** | **-** | **-** | **-** |
| 348 | male | 86 | urine | bilateral pneumonia | *E. coli* | I | S | **-** | **-** | **-** | **-** | **-** | **-** | **-** | **-** |
| 349 | male | 102 | sputum | pneumonia | *P. aeruginosa* | R | I | **+** | **-** | **-** | **-** | **+** | **-** | **-** | **-** |
| 350 | male | 95 | sputum | cerebral infarction sequelae | *A. baumannii* | R | R | **-** | **-** | **+** | **-** | **+** | **-** | **-** | **-** |
| 351 | male | 91 | sputum | bilateral pneumonia | *A. baumannii* | R | R | **-** | **-** | **+** | **-** | **+** | **-** | **-** | **-** |
| 352 | male | 90 | sputum | advanced gastric cancer | *P. aeruginosa* | R | R | **+** | **-** | **-** | **-** | **-** | **-** | **-** | **-** |
| 353 | male | 85 | sputum | cerebral infarction | *P. aeruginosa* | R | R | **+** | **+** | **-** | **-** | **-** | **-** | **-** | **-** |
| 354 | male | 87 | sputum | pneumonia | *P. aeruginosa* | R | R | **-** | **-** | **-** | **-** | **-** | **-** | **-** | **-** |
| 355 | male | 89 | sputum | pneumonia | *P. aeruginosa* | R | R | **+** | **-** | **-** | **-** | **+** | **-** | **-** | **-** |
| 356 | male | 87 | sputum | arrhythmia | *P. aeruginosa* | R | R | **+** | **-** | **-** | **-** | **-** | **-** | **-** | **-** |
| 357 | male | 93 | urine | bladder carcinoma | *E. coli* | R | R | **-** | **-** | **-** | **-** | **+** | **-** | **-** | **-** |
| 358 | male | 82 | sputum | pulmonary infection | *K. pneumoniae* | R | R | **+** | **-** | **-** | **-** | **-** | **-** | **-** | **-** |
| 359 | male | 87 | sputum | ventilator associated pneumonia | *K. pneumoniae* | R | R | **-** | **+** | **-** | **-** | **-** | **-** | **-** | **-** |
| 360 | male | 90 | sputum | hypertension | *P. aeruginosa* | R | R | **+** | **-** | **-** | **+** | **-** | **-** | **-** | **-** |
| 361 | male | 91 | sputum | lung cancer | *K. pneumoniae* | I | R | **+** | **-** | **-** | **-** | **-** | **-** | **-** | **-** |
| 362 | female | 98 | sputum | chronic renal failure | *P. aeruginosa* | R | R | **+** | **-** | **-** | **+** | **-** | **-** | **-** | **-** |
| 363 | female | 87 | sputum | acute cholecystitis | *P. aeruginosa* | R | R | **+** | **-** | **-** | **-** | **+** | **-** | **-** | **-** |
| 364 | male | 91 | sputum | cardiac insufficiency | *P. aeruginosa* | R | R | **-** | **-** | **-** | **-** | **+** | **-** | **-** | **-** |
| 365 | male | 94 | sputum | bilateral pneumonia | *P. aeruginosa* | R | R | **-** | **-** | **-** | **-** | **+** | **-** | **-** | **-** |
| 366 | male | 95 | sputum | radiation enteritis | *P. aeruginosa* | R | R | **+** | **-** | **-** | **-** | **+** | **-** | **-** | **-** |
| 367 | male | 90 | sputum | bilateral pneumonia | *P. aeruginosa* | R | R | **+** | **-** | **-** | **+** | **-** | **-** | **-** | **-** |
| 368 | male | 85 | sputum | bilateral pneumonia | *P. aeruginosa* | R | R | **+** | **-** | **-** | **-** | **-** | **-** | **-** | **-** |
| 369 | male | 90 | sputum | pulmonary infection | *P. aeruginosa* | R | R | **+** | **-** | **-** | **+** | **-** | **-** | **-** | **-** |
| 370 | male | 87 | urine | urinary infection | *A. baumannii* | R | R | **+** | **-** | **+** | **-** | **+** | **-** | **-** | **-** |
| 371 | male | 93 | sputum | acute cholecystitis | *P. aeruginosa* | R | R | **+** | **-** | **-** | **-** | **-** | **-** | **-** | **-** |
| 372 | male | 89 | sputum | atrophic gastritis | *P. aeruginosa* | R | R | **+** | **-** | **-** | **-** | **-** | **-** | **-** | **-** |
| 373 | male | 94 | sputum | pneumonia, hydrocephalus | *P. aeruginosa* | R | R | **+** | **-** | **-** | **+** | **-** | **-** | **-** | **-** |
| 374 | male | 93 | sputum | cerebral infarction sequelae | *P. aeruginosa* | R | R | **+** | **-** | **-** | **+** | **-** | **-** | **-** | **-** |
| 375 | male | 86 | sputum | Parkinson’s disease | *E. coli* | R | R | **+** | **-** | **-** | **-** | **-** | **-** | **-** | **-** |
| 376 | male | 88 | sputum | cerebral infarction sequelae | *A. baumannii* | R | R | **-** | **-** | **+** | **-** | **+** | **-** | **-** | **-** |
| 377 | male | 92 | sputum | COPD | *K. pneumoniae* | R | R | **+** | **-** | **-** | **-** | **-** | **-** | **-** | **-** |
| 378 | male | 95 | sputum | cerebral hemorrhage | *E. coli* | R | R | **+** | **-** | **-** | **+** | **-** | **-** | **-** | **-** |
| 379 | male | 83 | sputum | cerebral infarction | *A. baumannii* | R | R | **-** | **-** | **+** | **-** | **+** | **-** | **-** | **-** |
| 380 | male | 88 | sputum | pneumonia，respiratory failure | *K. pneumoniae* | R | R | **-** | **+** | **-** | **-** | **-** | **-** | **-** | **-** |
| 381 | male | 89 | sputum | cerebral hemorrhage | *A. baumannii* | R | R | **-** | **-** | **+** | **-** | **+** | **-** | **-** | **-** |
| 382 | female | 97 | sputum | bilateral pneumonia | *P. aeruginosa* | R | R | **-** | **-** | **-** | **-** | **+** | **-** | **-** | **-** |
| 383 | male | 85 | sputum | pneumonia | *P. aeruginosa* | R | R | **-** | **-** | **-** | **-** | **+** | **-** | **-** | **-** |
| 384 | male | 92 | sputum | gastric carcinoma | *A. baumannii* | R | R | **+** | **-** | **+** | **-** | **+** | **-** | **-** | **-** |
| 385 | male | 97 | sputum | diabetes mellitus type 2 | *P. aeruginosa* | R | R | **+** | **-** | **-** | **-** | **-** | **-** | **-** | **-** |
| 386 | male | 99 | sputum | pneumonia | *P. aeruginosa* | R | R | **+** | **-** | **-** | **-** | **-** | **-** | **-** | **-** |
| 387 | male | 86 | sputum | arrhythmia | *P. aeruginosa* | R | R | **+** | **-** | **-** | **+** | **-** | **-** | **-** | **-** |
| 388 | male | 97 | sputum | bilateral pneumonia | *P. aeruginosa* | R | R | **+** | **-** | **-** | **-** | **+** | **-** | **-** | **-** |
| 389 | male | 86 | sputum | pneumonia | *A. baumannii* | R | R | **-** | **-** | **+** | **-** | **+** | **-** | **-** | **-** |
| 390 | female | 86 | sputum | bilateral pneumonia | *P. aeruginosa* | I | R | **+** | **-** | **-** | **-** | **-** | **-** | **-** | **-** |
| 391 | male | 99 | sputum | bilateral pneumonia | *A. baumannii* | R | R | **-** | **-** | **+** | **-** | **+** | **-** | **-** | **-** |
| 392 | male | 82 | sputum | radiation enteritis | *P. aeruginosa* | R | R | **+** | **-** | **-** | **-** | **-** | **-** | **-** | **-** |
| 393 | male | 87 | sputum | pneumonia | *P. aeruginosa* | R | R | **+** | **-** | **-** | **-** | **-** | **-** | **-** | **-** |
| 394 | male | 88 | sputum | Parkinson’s disease | *P. aeruginosa* | R | R | **+** | **-** | **-** | **+** | **-** | **-** | **-** | **-** |
| 395 | male | 75 | sputum | angina pectoris | *P. aeruginosa* | R | R | **+** | **-** | **-** | **-** | **-** | **-** | **-** | **-** |
| 396 | male | 71 | sputum | bilateral pneumonia | *P. aeruginosa* | R | R | **+** | **-** | **-** | **-** | **-** | **-** | **-** | **-** |
| 397 | male | 83 | sputum | COPD | *P. aeruginosa* | R | R | **+** | **-** | **-** | **-** | **-** | **-** | **-** | **-** |
| 398 | male | 97 | sputum | hypertension (grade III) | *P. aeruginosa* | R | R | **+** | **-** | **-** | **+** | **-** | **-** | **-** | **-** |
| 399 | male | 96 | sputum | pneumonia | *K. pneumoniae* | R | R | **+** | **-** | **-** | **-** | **-** | **-** | **-** | **-** |
| 400 | male | 80 | sputum | intrahepatic cholangiocarcinoma | *E. coli* | R | R | **-** | **-** | **-** | **-** | **+** | **-** | **-** | **-** |
| 401 | male | 91 | sputum | pneumonia | *A. baumannii* | R | R | **-** | **-** | **+** | **-** | **+** | **-** | **-** | **-** |
| 402 | male | 88 | sputum | cerebral infarction sequelae | *A. baumannii* | R | R | **-** | **-** | **+** | **-** | **+** | **-** | **-** | **-** |
| 403 | male | 78 | sputum | pneumonia，gastrointestinal dysfunction | *P. aeruginosa* | R | R | **-** | **-** | **-** | **-** | **+** | **-** | **-** | **-** |
| 404 | male | 86 | sputum | bilateral pneumonia | *P. aeruginosa* | R | R | **+** | **-** | **-** | **-** | **+** | **-** | **-** | **-** |
| 405 | male | 87 | sputum | chronic heart failure | *P. aeruginosa* | R | R | **+** | **-** | **-** | **-** | **-** | **-** | **-** | **-** |
| 406 | male | 85 | sputum | pneumonia | *P. aeruginosa* | R | R | **-** | **+** | **-** | **-** | **-** | **-** | **-** | **-** |
| 407 | male | 88 | sputum | advanced lung cancer | *P. aeruginosa* | R | R | **+** | **-** | **-** | **-** | **-** | **-** | **-** | **-** |
| 408 | female | 93 | sputum | Parkinson’s disease | *P. aeruginosa* | R | R | **+** | **-** | **-** | **-** | **-** | **-** | **-** | **-** |
| 409 | male | 82 | sputum | bilateral pneumonia | *A. baumannii* | R | R | **-** | **-** | **+** | **-** | **+** | **-** | **-** | **-** |
| 410 | male | 91 | sputum | radiation enteritis | *P. aeruginosa* | R | R | **-** | **-** | **-** | **+** | **-** | **-** | **-** | **-** |
| 411 | male | 84 | urine | urinary infection | *E. coli* | R | R | **+** | **-** | **-** | **-** | **-** | **-** | **-** | **-** |
| 412 | male | 83 | sputum | bilateral pneumonia | *E. coli* | R | R | **+** | **-** | **-** | **-** | **-** | **-** | **-** | **-** |
| 413 | male | 84 | sputum | pulmonary infection | *P. aeruginosa* | R | R | **+** | **-** | **-** | **-** | **-** | **-** | **-** | **-** |
| 414 | male | 90 | sputum | cerebral infarction sequelae | *P. aeruginosa* | R | R | **+** | **-** | **-** | **+** | **-** | **-** | **-** | **-** |
| 415 | male | 82 | sputum | cerebral infarction sequelae | *E. coli* | R | R | **-** | **-** | **-** | **-** | **+** | **-** | **-** | **-** |
| 416 | female | 95 | sputum | coronary artery disease | *P. aeruginosa* | R | R | **+** | **-** | **-** | **-** | **-** | **-** | **-** | **-** |

Note: IMP, imipenem; MEM, meropenem ; KPC, *Klebsiella pneumoniae* carbapenemases; NDM-1, New Delhi metallo-β-lactamase; OXA-23, oxacillinase-23; OXA-48, oxacillinase-48 ;OXA-51, oxacillinase-51; IMP , Imipenem metallo-β-lactamase; VIM, Verona integron-encoded metallo-β-lactamase; DIM; Dutch imipenemase; R, resistant; S, sensitive; **+**, positive; **-**, Negative
